# Optimisation of the cultured ELISpot/Fluorospot technique for the selective investigation of SARS-CoV-2 reactive central memory T cells

**DOI:** 10.3389/fimmu.2025.1547220

**Published:** 2025-04-15

**Authors:** Jack R. Jerome, Kirsty L. Wilson, Joshuah Fialho, Georgia Goodchild, Monica D. Prakash, Charlie McLeod, Peter C. Richmond, Vasso Apostolopoulos, Katie L. Flanagan, Magdalena Plebanski

**Affiliations:** ^1^ School of Health and Biomedical Science, Royal Melbourne Institute of Technology University, Melbourne, VIC, Australia; ^2^ Accelerator for Translational Research in Clinical Trials (ATRACT) Centre, School of Health and Biomedical Sciences, Royal Melbourne Institute of Technology University, Melbourne, VIC, Australia; ^3^ Wesfarmers Centre of Vaccines and Infectious Diseases, Kids Research Institute of Australia, Perth, WA, Australia; ^4^ Centre for Child Health Research, University of Western Australia, Perth, WA, Australia; ^5^ Sydney School of Public Health, Faculty of Medicine and Health, University of Sydney, Sydney, NSW, Australia; ^6^ Infectious Diseases Department, Perth Children’s Hospital, Perth, WA, Australia; ^7^ Division of Paediatrics, University of Western Australia School of Medicine, Perth, WA, Australia; ^8^ Department of Immunology, Perth Children’s Hospital, Perth, WA, Australia; ^9^ Tasmanian Vaccine Trial Centre, Clifford Craig Foundation, Launceston General Hospital, Launceston, TAS, Australia; ^10^ School of Health Sciences and School of Medicine, University of Tasmania, Launceston, TAS, Australia

**Keywords:** ELISpot, Fluorospot, method, SARS-CoV-2, COVID-19, COVID, central memory T-cell responses, immune responses

## Abstract

**Introduction:**

This study presents an optimised cultured ELISpot protocol for detecting central memory T-cell interferon gamma (IFNγ) responses against SARS-CoV-2 peptides following an initial priming with either peptides, or whole spike protein.

**Methods:**

Key variations optimised include the culture length, timing of exogenous survival signals (IL-2), and endpoint analysis modality and cell density to enhance assay sensitivity without compromising specificity for central memory T-cell IFNγ recall responses to cognate antigen.

**Results:**

We noted a culture duration of 10 days, combined with a delayed IL-2 administration on day 5 to enhance assay sensitivity while maintaining response specificity towards cognate antigen when compared with shorter culture periods or earlier exogenous survival signal provision. With regards to lower-frequency T-cell interactions, as we observed with our donor SARS-CoV-2 epitope responses, our findings suggest Fluorospot to be preferable to the chromogenic ELISpot modality, and an immediate cell washing after culture collection to better facilitate cognate antigen responses. Fluorospot enabled a higher cell density while minimising the generation of visual artefacts, meanwhile immediate cell washing was critical for improving endpoint assay sensitivity. CCR7+ cell depletion was used to demonstrate our optimised protocol to selectively demonstrate central memory T-cell responses. Lastly, we provide evidence for the capacity of our assay to delineate individual responding peptides following peptide pool priming, and to explore cross-reactivity between viral variant peptides.

**Conclusion:**

This work advances the methodology for investigating T-cell immunity, particularly in the context of SARS-CoV-2, and emphasises the balance between enhancing specific cognate central memory responses while limiting non-specific activation.

## 1 Introduction

As vaccination strategies seek to ensure long-lasting protection, analysing the memory T-cell compartment becomes crucial for understanding immune responses and optimising future vaccine development and effectiveness (1, 2). Memory T cells, sub-categorised into tissue-resident and circulating memory T cells, provide a cellular compartment of immunological memory (3, 4). Circulating memory T cells are sub-categorised into effector and central memory T cells (1). C–C Chemokine receptor type 7 (CCR7) expression on central memory T cells permits their migration between lymphoid organs through the vasculature, whereas effector memory T cells survey peripheral organs in addition to tissue-resident memory T cells (3, 4) providing robust recall responses to their cognate antigen during repeated infection or exposure post-vaccination. Both T-cell compartments, effector and central memory, can be analysed respectively through the functional assays, *ex-vivo* Enzyme-Linked Immunospot (ELISpot) and Fluorospot, or the cultured ELISpot method (5–7). The immediate provision of recall antigen in *ex-vivo* ELISpot preferentially investigates effector T cells (5), whereas the antigen-primed culture period of cultured ELISpot assays favours the preferential proliferation of central memory T cells, generating an expanded pool of cognate cells able to respond rapidly by cytokine secretion to subsequent challenges (8–10). Cultured ELISpot techniques enhance the expansion of central memory T cells by 1) culturing cells for 8–14 days before analysis and 2) providing exogenous survival signals such as interleukin (IL)-2. Delaying administration of IL-2 allows for the elimination of non-cognate adaptive cells and many innate immune cells, while promoting the preferential proliferation of antigen-stimulated central memory T cells (8–10). Several studies have investigated the relationship between *ex-vivo* and cultured ELISpot T-cell responses. Flanagan et al. initially proposed that antigen-specific *ex-vivo* and cultured ELISpot responses do not necessarily predict one another (11), with subsequent studies demonstrating central memory T cells, but not effector memory T cells, to drive cultured ELISpot responses to T-cell epitopes correlated with protection against malaria, whereas *ex-vivo* ELISpot responses were not (12). Subsequently, no correlation was noted between *ex-vivo* and cultured ELISpot interferon gamma (IFNγ) responses towards the hepatitis C virus genotype 1a peptides (5). However, with reference to acute infections, *ex-vivo* and cultured ELISpot responses appear comparable around 7 days post-infection, whereas over time, the ratio of cultured ELISpot to *ex-vivo* responses shows a substantial increase (7). These studies highlight the necessity for robust techniques to separately study effector T-cell and central memory T-cell compartments.

The spike (S) protein of SARS-CoV-2 is broadly sub-categorised into two subunits, S1 and S2, with the S1 region containing the N-terminal domain (residues 14–205) and the receptor-binding domain (residues 319–541) that facilitates angiotensin-converting enzyme 2 (ACE-2) recognition (13). The spike protein and its ACE-2-binding domain are predominantly targeted by vaccines aiming to induce antibodies to block this interaction and prevent cell recognition and entry (14). T cells have been recognised for their role in providing directly to protective immunity against SARS-CoV-2, with a link between a deficiency of IFNγ-producing CD4^+^ T cells, and weaker neutralising antibody responses to SARS-CoV-2 and higher risk of hospitalisation (15). Additionally, T follicular helper cells indirectly assist with SARS-CoV-2 responses through supporting B-cell maturation (16). Although significant literature exists on measuring circulating effector CD4^+^ T cells reactive to SARS-CoV-2 proteins post-infection or vaccination, particularly through *ex-vivo* ELISpot (17–19), there is limited understanding of how long-term central memory CD4^+^ T-cell reservoirs are generated. These reservoirs are essential for rapid activation and expansion in responses to future antigenic challenges. Indeed, while it is acknowledged that the induction and persistence of central memory T cells is key to the establishment of long-term protection against a wide variety of diseases, there is no harmonised assay to measure central memory functionally for direct comparison with the effector T-cell compartment in a high-throughput manner. Flow cytometry and tetramer-based assays are labour intensive and require substantial cell quantities, making them challenging to use as a high-throughput method to measure multiple T-cell epitopes or assessing broad functional cross reactivity (20, 21). The leading central memory functional assay, the cultured ELISpot, is capable of offering high-throughput analysis and has been used to study central memory immunity across cancer (22), viral diseases (23), and parasitic diseases (11, 12). However, cultured ELISpot protocols vary significantly between laboratories, with no systematic investigation for their implementation in analysing SARS-CoV-2 cellular immune responses (**Table 1**). By its nature, the cultured ELISpot technique generates a myriad of variables to fine-tune to quantify cognate antigen responses. Using SARS-CoV-2 antigens, we investigate these variables and outline a comprehensive protocol designed to elicit central memory immune responses to specific SARS-CoV-2 proteins or peptides. This protocol demonstrates the capacity to map diverse epitopes and assess cross-reactivity between viral variants through the prime, culture, and restimulation phases inherent to the cultured ELISpot technique. We further show that combining cultured ELISpot with modern Fluorospot analysis enhances the assay’s capability to detect central memory immune responses. Herein, we assess and optimise the cultured ELISpot assay for the study of central memory T-cell immune responses against the S1 region of SARS-CoV-2.

**Table 1:** Diversity within published cultured ELISpot protocols.

Culture variables			Survival stimulant variables		Endpoint variables		
Culture length	Prime stimulant	Washing/resting	Days of provision	Stimulant, concentration	Endpoint ELISpot cells/well	Endpoint analysis	Reference
14 Days	25 µg/mL/peptide 15-mer Circumsporozoite protein peptides	Washed 3 times after harvest	Days 5, and 10	Lymphocult-T, 10 U/mL	10K cells/well	Correlated *ex vivo* and cultured ELISpot with proliferation assays Whole protein and peptide priming	Pinder et al. (2004) (39)
14 Days	25 µg/ml/peptide 15-mer Circumsporozoite protein peptides	Washed once after harvest	Days 5, and 10	Lymphocult-T, 10 U/mL	20K cells/well	Correlated post-vaccination cultured ELISpot responses with subsequent malaria parasitemia, and protection Cultured ELISpot time course for IFNγ release after vaccination	Reece et al. (2004) (12)
14 days	25 µg/mL/peptide 15-mer overlapping Circumsporozoite protein peptides	Washed once after harvest	Days 5 and 10	IL-2, 0IU/mL	10K cells/well	Compared sensitivity of *ex vivo* and cultured ELISpot Correlated *ex vivo and* cultured ELISpot with antibody levels, proliferative assays, and blood smear positivity (malaria)	Flanagan et al. (2001) (11)
12 days	4 µg/mL peptide pools in 100 µL for 1 h at 37°C, before twofold dilution 15-mer overlapping by 11 amino acid peptide pools covering HBV genotypes B and C	Washed on day 10, rested for 36 hours	Day 0 Days 3, 7, and 8	rhIL-7, 25ng/mL rhIL-2, 10 or 100 ng/mL	20K cells/well	Correlated *ex vivo* and cultured ELISpot HBV responses Compared IL-2 concentration of assay sensitivity	Chen et al. (2021) (40)
12 days	10 µg/mL peptide 15-mer non-overlapping Hepatitis C Virus peptides	Washed three times and immediately assayed	Days 3, 6, and 9	Lymphocult T, 10%	25K cells/well	Correlated *ex vivo* and cultured responses over 24 months CCR7, and CD8 depletion of cultured ELISpots	Godkin et al. (2002) (5)
10 days	200 µL of 40 µM DENV serotype peptides	Wash and rest for 1-2 days	Days 3 and 7	IL-2, 100 IU/mL	40K cells/well	Dengue virus serotype-specific responses	Jeewandara et al. (2018) (23)
10 days	EBV peptide pools 15-mer overlapping by 11 amino acids peptide pools covering several EBV proteins	Washed three times after harvest	Days 3 and 7	rhIL-2, 20 IU/mL	4K cells/well	Comparative *ex vivo* and cultured ELISpot EBV responses Cultured ELISpot quantitative reproducibility CD4, and CD8 depletion in cultured ELISpot	Calarota et al. (2013) (6)
10 days	5–10 µg/mL of peptides 57 20-mer overlapping by 10 amino acids peptides covering TRAP protein	Washed three times on day 9, rested overnight.	Days 3 and 7	IL-2, 50 U/mL	n/a	Correlated *ex vivo* and cultured ELISpot responses in unvaccinated or prime-boosted individuals Time course characterisation of IFNγ SFC, cognate antigen tetramer staining, memory marker staining CCR7, and CD62L depletion on *ex vivo* and cultured ELISpot	Todryk et al. (2009) (7)
10 days	10 µg/mL/peptide of 57 20-mer overlapping by 10 amino acids covering the TRAP peptides	Washed three times on day 9, overnight rest, washed again	Days 3 and 7	Lymphocult, 10 IU/mL	Approx 25K cells/well	TRAP memory T-cell responses	Keating et al. (2005) (38)

## 2 Materials and methods

### 2.1 Study population

Six blood samples containing concentrated white blood cells (buffy coat) from donors were supplied by the Australian Red Cross Lifeblood (RMIT HREC #21681). Whole blood was collected by the Cancer Ageing and Vaccines Research Group from one local donor (Melbourne, Australia) as approved by the RMIT Human Research Ethics Committee (Ethics #: 24280). Blood donors were randomly spread across sex and age with 57% being female (4/7) with a median age of 57 years and IQR of 30.5 years and randomly assigned a deidentified identifier from A to G (**Supplementary Table S1**). The HLA haplotype was not assessed for donors, and information regarding prior SARS-CoV-2 exposure or vaccination history was not available for Australian Red Cross Lifeblood donors. Donor A was included across all experiments with additional replicates spread across donors B–G (as defined in **Supplementary Tables S2**-**S8**). Donor data were collected and managed using REDCap (RRID: SCR_003445) electronic data capture tools hosted at RMIT University (24, 25).

### 2.2 Isolation of PBMCs from buffy coats and whole blood

In sterile conditions, buffy coat samples were diluted 3:2 with RPMI 1640 (no L-glutamine, Gibco, 21870100). Whole blood was collected from the volunteer in EDTA vacutainer tubes (BD Vacutainer, BD, USA). Either buffy coat or fresh whole blood were processed via the same protocol detailed below. Whole-blood or RPMI-diluted buffy coat was carefully overlaid at a 45° angle onto 10 mL of Ficoll-Plaque PLUS (GE Healthcare, GEHE17-1440-03) and centrifuged at 2,000 RPM for 20 min at room temperature with half-speed acceleration and no brake. The PBMC layer was carefully collected and washed with RPMI (centrifugation at 1,400 RPM for 4 min at room temperature, brake on). Samples underwent red blood cell removal (RBC lysis buffer, BioLegend, 420301) for 5 min before a second wash. Pellets were resuspended in RPMI supplemented with 5% heat-inactivated human serum (Sigma, H4522-100mL) before counting with Trypan blue (Gibco, 15250-061) in duplicate using a Countess 3 FL Cell Counter (Thermo Fisher). RPMI and human serum were removed and PBMCs resuspended in human serum with 10% DMSO and frozen gradually with the Mr Frosty (Thermo Scientific, 5100-0001) before long-term storage in LN2.

### 2.3 Peptides and proteins

**Figure 1:**
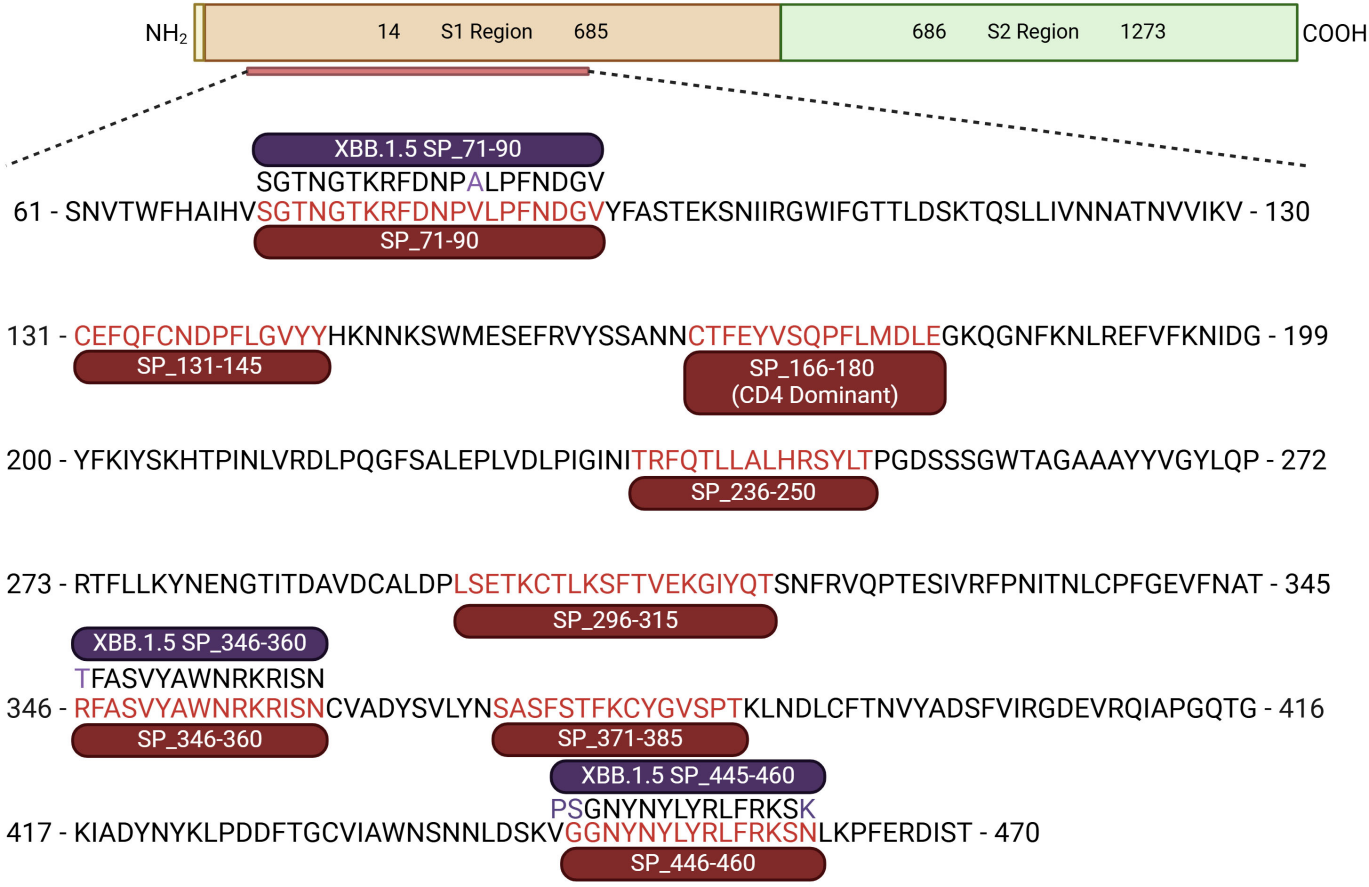
SARS-CoV-2 S1 peptide pool and overlapping XBB variants. Schematic representation of the SARS-CoV-2 spike protein (1–1,273 amino acids). Defining S1 (residues 14–685), and S2 (residues 686–1273) regions. Sub-section of the S1 region containing eight 2019-nCOVID S1 peptide sequences, including the CD4 dominant peptide (SP_166-180) consisting of the S1 peptide pool. Three overlapping variant XBB.1.5 peptides are also shown with their respective S1 peptides; mutations are highlighted purple (55).

The Immune Epitope Database and Tools resource (IEDB, RRID: SCR_006604) (26) was used to initially investigate experimentally validated IFNγ-stimulating epitopes within the Wuhan SARS-CoV-2 (ID: 2697049) spike glycoprotein (UniProt: P0DTC2). Peptide regions within the spike glycoprotein subunit 1 between 15 and 20 amino acids in length were then investigated *in silico* with the IEDB MHC II Binding T Cell Epitope Prediction against a list of common Australian HLA haplotypes (**Supplementary Table S9**) defined in the Allele Frequencies in Worldwide Populations Database (RRID: SCR_007259) (27). Additional peptides were cross-checked and selected from the literature (28). The predicted rank, score, and capacity to bind multiple common Australian HLA haplotypes were considered when selecting peptide sequences for inclusion in our S1 peptide pool; in total, eight peptides were selected across the S1 region (**Figure 1**). SARS-CoV-2 XBB.1.5 mutations were obtained from the GISAID COVID-19 mutation dashboard (29), with viral variant peptides generated from aligning mutational variants overlapping individual S1 peptides in the S1 peptide pool. The XBB S1 overlapping peptide pool contained the following three peptides at a pooled concentration of 50 µg/mL: 1) XBB SP_71-90 (SGTNGTKRFDNPALPFNDGV), 2) XBB SP_346-360 (TFASVYAWNRKRISN), and 3) XBB SP_455-460 (PSGNYNYLYRLFRKSK) (**Figure 1**). All peptides were synthesised to 95% purity, with free ends by Mimotopes (Clayton, VIC Australia). Conditions requiring NaOH or DMSO to facilitate peptide solubility are outlined in **Supplementary Table S10**. Whole 2019-nCoV Spike protein was purchased from Sino Biological (>90% purity, 40589-V08B1) for the investigation of whole protein priming in cultured ELISpot. A known strong IFNγ-inducing cytomegalovirus (CMV) peptide, CMV-495 (NLVPMVATV) (30), was used as a housekeeper peptide for initial experiment optimisations. Purified protein derivative (PPD, AJ Vaccines) was used as a known strongly responsive recall antigen to investigate assay specificity through providing a comparable non-cognate recall response.

### 2.4 Thawing of frozen PBMCs

PBMC vials were briefly thawed in a water bath at 37°C and then transferred to a BSCII and diluted with complete RPMI (RPMI 1640 [Gibco, 21870076] supplemented with L-glutamine [2% v/v 200 mM, Gibco, A2916801], penicillin–streptomycin [1% v/v 10,000 U/mL, Gibco, 15140-122], heat-inactivated human serum [5% v/v, Sigma, H4522-100ML], and hepes [2% v/v, Gibco, 15630-080]). Following centrifugation at 700 g for 7 min at room temperature, supernatant was discarded, and cells were resuspended in fresh complete RPMI before centrifugation at 1,400 RPM for 4 min at room temperature. PBMCs were resuspended in fresh complete RPMI before counting on the Countess 3 FL Cell Counter, diluted to 5 × 10^6^ cells/mL, and rested for 3 h at 37°C in a humidified incubator with 5% CO_2_. Post-rest, cells were passed through a 100-µm cell strainer to remove debris, washed, and resuspended in complete RPMI for counting and resuspension for downstream analysis according to the specified cells/mL required for *ex-vivo* and cultured ELISpot analysis, respectively (refer to below sections).

### 2.5 Cultured ELISpot cell culture

**Figure 2:**
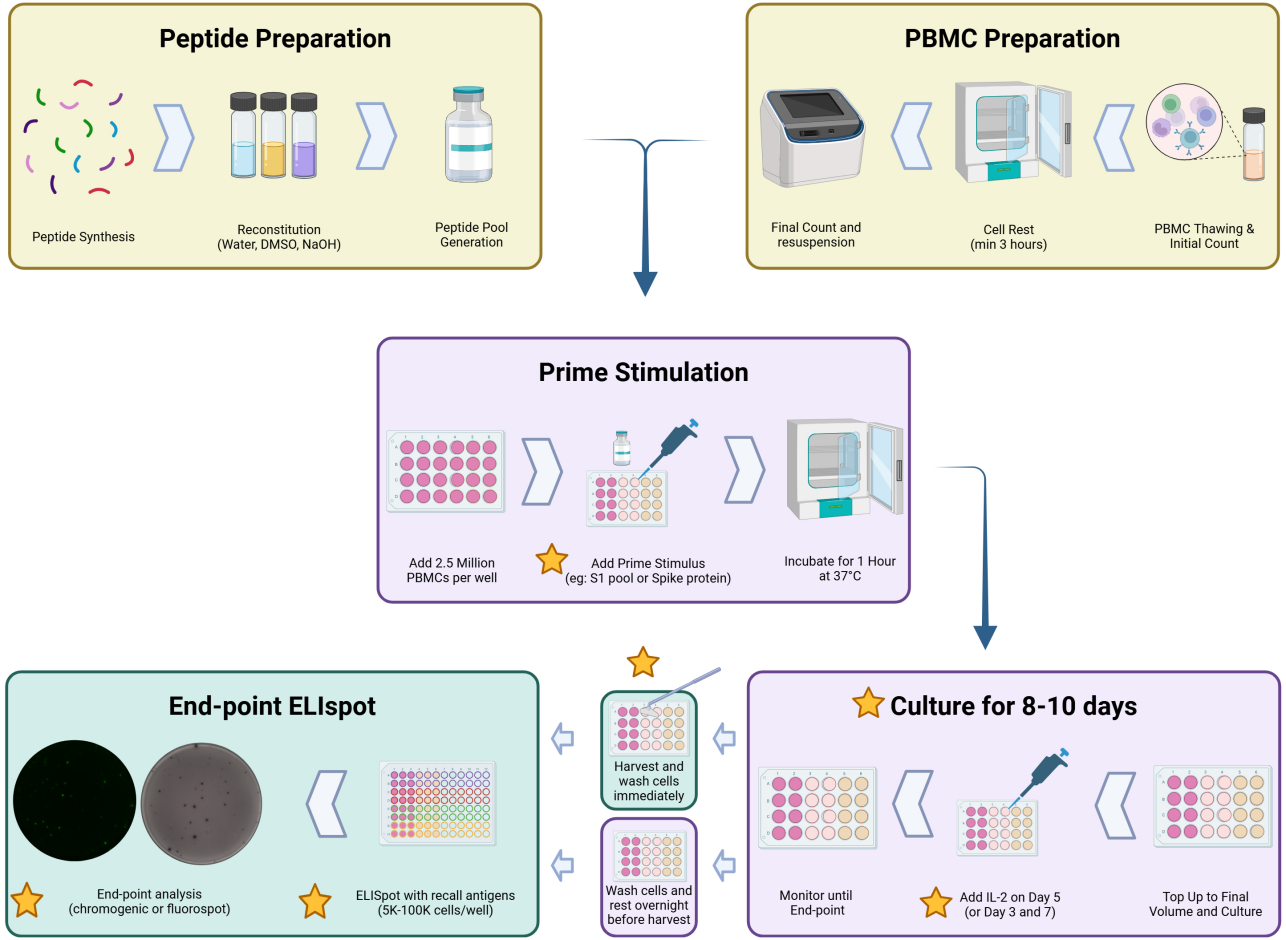
Graphical methodology of the cultured ELISpot protocol. Yellow stars indicate the areas of optimisation discussed in this paper (56).

**Figure 3:**
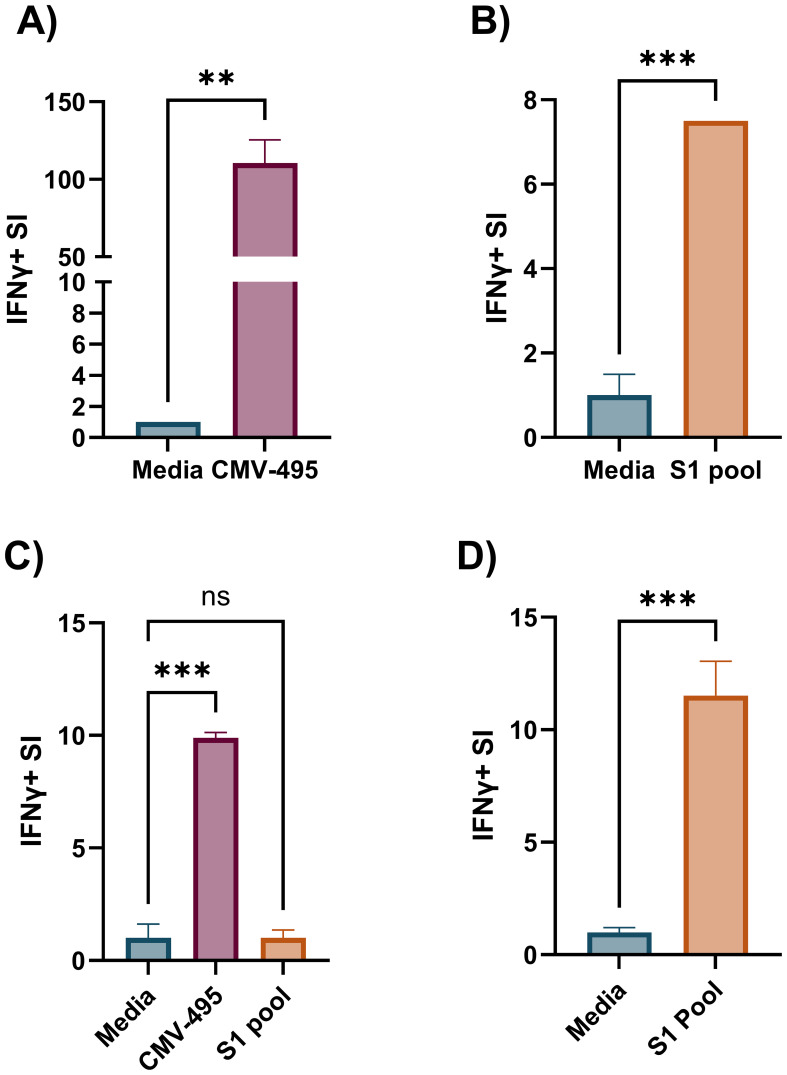
Increased cultured ELISpot assay sensitivity for less robust antigens requires increased cell numbers tested in a fluorogenic assay. IFNγ stimulation index (SI) to media and cognate antigen in *ex-vivo* ELISpot with 350 × 10^3^ cells/well for **(A)** CMV-495 and **(B)** S1 pool. **(C)** Cultured ELISpot analysis of PBMCs primed to either CMV-495 or S1 peptide pool and cultured for 10 days before re-exposure to media or cognate antigen: CMV-495 or S1 pool, using 10 × 10^3^ cells/well chromogenic ELISpot, **(D)** or to S1 pool using 100x10^3^ cells/well in Fluorospot ELISpot. Graphs present the mean ± standard deviation (SD) of SI of one representative donor [donor A, **Supplementary Tables S1, S2–S4**] of two-three donors analysed in duplicate-triplicate. **(A, B, D)** Unpaired *t*-test, and **(C)** one-way ANOVA were used for statistical significance and depicted as **p < 0.01, ***p < 0.005; ns, not significant.

To set up cultured ELISpot, PBMCs were thawed as above and plated immediately on day 1 of the time course. PBMCs were plated in a sterile 48-well flat-bottom plate (CELLSTAR, 677-180) at 2.5 × 10^6^ cells/well in 250 µL of complete RPMI and primed with 1) Wuhan SARS-CoV-2 S-protein (50 µg/mL, Sino Biological, 40589-V08B1), 2) S1 peptide pool (peptides each at a concentration of 50 µg/mL, **Figure 1**), or 3) CMV-495 (50 µg/mL). Cells were primed for 1 h at 37°C in a humidified incubator before topping each well up to 1 mL with complete RPMI. Stimulants specifically used for both priming and later downstream restimulation were all used at 50 µg/mL. Wells were monitored for a yellow media colour indicative of a low pH and subsequent nutrient depletion, upon which a media change involving the careful aspiration of 500 µL of culture media and supplementation with 500 µL of fresh and warmed complete RPMI. In the media supplementation on day 5 of culture, 10 IU of recombinant human IL-2 (Thermo Fisher Scientific, PCH0021) per 500 µL was included unless otherwise specified in the Results section. Unless otherwise specified, end-point analysis occurred after 10 days of culture where PBMCs were collected from the plate, washed, counted, and resuspended to 2 × 10^6^ cells/mL and set up as per the *ex-vivo* ELISpot assay detailed below, with varied PBMC numbers/well as outlined in each respective figure description. Samples that underwent an overnight wash were washed once and resuspended in 1 mL of complete RPMI and rested overnight before analysis in Fluorospot ELISpot (**Figure 2**).

### 2.6 Chromogenic ELISpot and Fluorospot

Both *ex-vivo* chromogenic ELISpot and cultured ELISpot endpoint analysis with chromogenic or Fluorospot plates followed the same protocol. Antigen-specific CD4 T-cell effector IFNγ responses were evaluated with chromogenic ELISpot. 96-well filtration plates (MSIP plates, MSIPS4510, Millipore, Billerica, MA USA) were activated with 15 µL/well of 35% ethanol for less than 1 min, washed five times with 200 µL/well of PBS, and coated with 100 µL/well of anti-human IFNγ unconjugated monoclonal antibody (mAb) (5 µg/mL, 1-DIK, MAB3420-3-1000, Mabtech, Sweden) overnight at 4°C. Plates were then washed five times with PBS at room temperature and blocked with complete RPMI for 1–2 h at 37°C. Thawed PBMCs were resuspended to 0.1–2 × 10^6^ cells/mL (depending on culture conditions stated in Figure legends), and 50 µL was added in singlicate to triplicate with 50 µL of recall antigens to a final concentration of 50 µg/mL: CMV-495, S1 pool (all peptides each at a final concentration of 50 µg/mL), SP_131-145, SP_166-180, XBB S1 overlapping pool (all peptides each at a final concentration of 50 µg/mL), or 25 µg/mL of purified protein derivative (PPD) (31) before incubation for 16 h for chromogenic, or 18 h for Fluorospot at 37°C in a humidified incubator with 5% CO_2_. CD4 peptides have previously been shown to require final concentrations between 25 and 100 µg/mL for efficient ELISpot analysis (12, 32–34). Due to our endpoint investigation of potentially less dominant and cross-reactive responses, we used peptides at a higher prior validated concentration of 50 µg/mL. Anti-CD3 (0.5 µg/mL, Mabtech) was used as a positive control. As some peptides required reconstitution with additive DMSO or NaOH, control wells containing media alone, media with 0.49% v/v 1 M NaOH, or media with 0.375% v/v DMSO were used as background controls (averaged and graphed as “Media”). NaOH and DMSO background concentrations were selected to match potential maximal concentrations, and all conditions assessed contained less solvents than present in the background controls (**Supplementary Table S10**). Plates were washed for 5 min with ultrafiltered reverse osmosis water (Milli-Q) to lyse cells before washing five times with PBS and incubating with anti-human IFNγ biotinylated mAb (1 µg/mL MAB-3420-6-1000, Mabtech, Sweden) at room temperature, protected from light, for 2 h. After washing five times with PBS, streptavidin-alkaline phosphatase was added to a final concentration of 1 µg/mL and incubated at room temperature, protected from light, for 1.5 h. Plates were washed five times with PBS, and then with Milli-Q, and spots were developed with a colorimetric AP kit (Bio-Rad, Philadelphia, USA) following the manufacturer’s instructions. Antigen-specific CD4 T-cell effector and central memory T-cell IFNγ and IL-10 responses were evaluated with IFNγ/IL-10 Fluorospot. Fluorospot assays were run as per manufacturer’s protocol (IFNγ/IL-10 plates X-01A07B-10, Mabtech, Sweden) with an 18-h incubation at 37°C in a humidified incubator with 5% CO_2_. For both the chromogenic ELISpot and Fluorospot, dry plates were imaged, and spots were counted with AID Multispot System software (v 7.0, AID, Straβberg, Germany).

### 2.7 Cultured ELISpot on CCR7+-depleted cell fractions

For depletion experiments, samples were split after resting the cells into non-depleted and depleted experimental wells. Briefly, non-depleted wells were set up and cultured as described above at 2.5 × 10^6^ cells/well. Depleted samples were generated by resuspending in 800 µL of FACS buffer (PBS with 5% v/v heat-inactivated FBS: Sigma-Aldrich,18J032) per 10^8^ cells with CCR7-PE antibody (552176, BD Pharmingen) for 15 min at room temperature with light protection. Cells were centrifuged at 1,400 RPM for 4 min at room temperature, resuspended with 800 µL Miltenyi buffer (PBS with 2 mM EDTA, and 0.5% BSA w/v, Bovogen, BSAS 0.1) per 10^8^ cells and 200 µL/10^8^ cells of anti-PE negative selection microbeads (Miltenyi Biotec, 120-000-294), and incubated for 15 min at 4°C. The cells were washed in Miltenyi buffer with 10–20 times the labelling volume before centrifugation at 300xg for 10 min. Cells were resuspended in 1 mL of Miltenyi buffer, and an LD column (Miltenyi Biotech, 130-042-961) was prepared with a 3-mL wash of Miltenyi buffer. Cells were then run through the column on the magnetic field MACS separator, after which the column was washed twice with Miltenyi buffer. Pre- and post-MACS separation, cell fractions were collected for subsequent flow cytometry analysis and CCR7 depleted cells were set up for cultured ELISpot at 2.5 × 10^6^ cells/well as per non-depleted cells.

### 2.8 Flow cytometry

CCR7 depletion was validated by flow cytometry of the following cell factions: 1) pre-MACS separated, 2) flow-through, and 3) cells collected within the LD column (**Supplementary Figure S1**, **Supplementary Table S11**). Unlabelled pre-MACS cells were labelled as per above with the PE-CCR7 antibody and then transferred to a 96-well v-bottom plate and centrifuged at 1,400 RPM, 4°C for 4 min; supernatants were removed by flicking the plate; and cells were washed with 150 µL of FACS buffer and centrifuged again. Cells were resuspended in 30 µL/well of Zombie Aqua (BioLegend, 423102, 1:1,000 dilution) and incubated with light protection at room temperature for 15 min. Cells were then washed, resuspended in 100 µL/well of PBS containing 1% paraformaldehyde, and then transferred to microtubes for analysis with a Fortessa X-20 benchtop (BD). Cytometry results were analysed using FlowJo™ v10.8 Software (BD Life Sciences, RRID: SCR_008520).

### 2.9 Data analysis and statistics

Data are presented as stimulation index (SI). SI calculations divide all individual responses including the various media conditions by their respective unstimulated average background responses to adjust for the variable background reactivity of individual donors. Where individualised data are required, such as in our investigation of individual peptide responses, and cross-reactivity between reference and XBB.1.5 viral variant peptides, spot forming units (SFU) per million PBMCs (SFU/×10^6^ cells) are presented. All Figures (unless otherwise stated) depict one representative donor’s responses, with all individualised donor data available in **Supplementary Tables S2**-**S8**. As indicated, unpaired *t*-test, one-way ANOVA, and two-way ANOVA were used for statistical analysis. Graphs were generated and statistically analysed using GraphPad Prism v.10.3.1. Data were analysed against the null hypothesis, with a statistically significant rejection of the null hypothesis considered at p ≤ 0.05.

## 3 Results

### 3.1 Varying cell input numbers and *ELISpot* readout modality from chromogenic to fluorescent, to enable detection of SARS-CoV-2 S1 central memory T-cell responses

**Figure 4:**
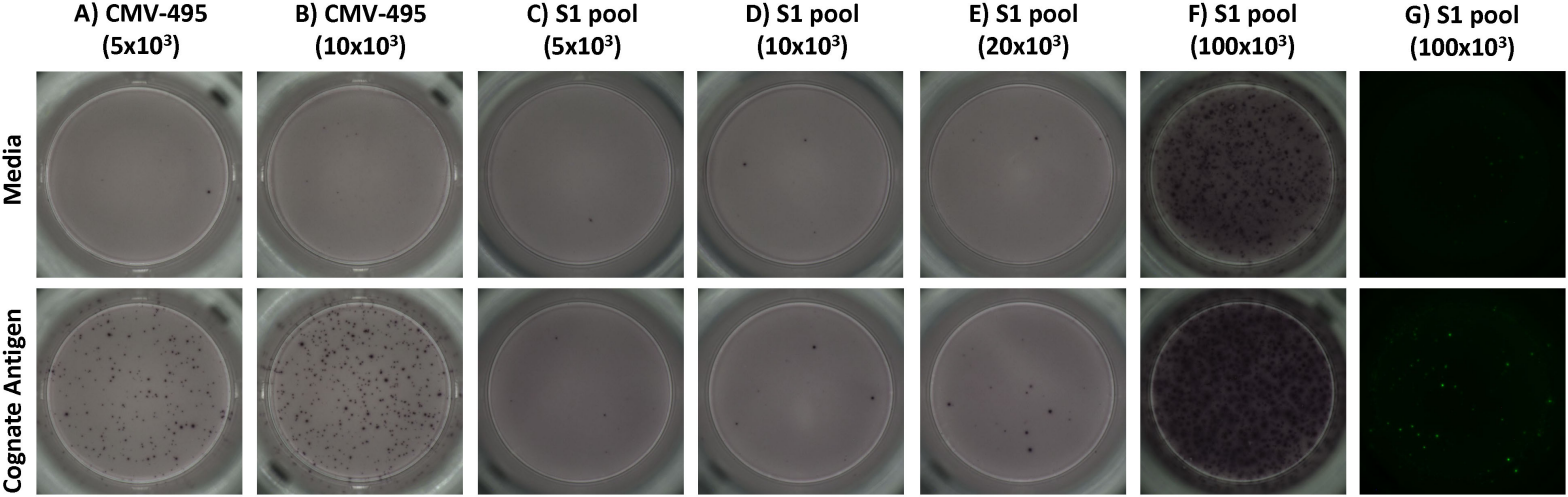
The fluorescent ELISpot modality, Fluorospot, enables the required specificity and sensitivity for SARS-CoV-2 antigen IFNγ recall responses. Representative images of recall IFNγ responses to unstimulated (media) and prime antigen matched restimulation (cognate antigen) for **(A, B)** CMV-495, or **(C-G)** S1 pool. CMV-495 recall responses were observed at either **(A)** 5 × 10^3^ or **(B)** 10 × 10^3^ cells/well. Chromogenic analysis with cell/well densities of **(C)** 5 × 10^3^, **(D)** 10 × 10^3^, or **(E)** 20 × 10^3^ did not have the required sensitivity to demonstrate S1 pool IFNγ recall responses, meanwhile **(F)** 100 × 10^3^ cells/well increased background visual artefacts. **(G)** Fluorescent ELISpot (Fluorospot) enhanced assay sensitivity and specificity to separate S1 pool IFNγ recall responses from background responses without visual artefacts with cells at 100 × 10^3^ per well.

Effector memory T-cell responses by *ex-vivo* ELISpot were initially used to screen donors for their potential corresponding central memory T-cell responses by cultured ELISpot. For this screening, we chose a broadly known T-cell epitope from cytomegalovirus (CMV-495) known to elicit strong IFNγ-producing effector T-cell responses in humans (35, 36), with a 57% seropositive rate for Australian adults (37), and a pool of experimentally validated (28) and predicted peptides capable of binding common Australian HLA haplotypes (**Supplementary Table S9**) corresponding to key T-cell epitopes from the SARS-CoV-2 S1 region (S1 pool). Generally, CMV-495 elicited robust effector (**Figure 3A**) and central memory responses (**Figure 3C**), which could be detected using as little as 5 × 10^3^ cells/well in the subsequent chromogenic ELISpot assay following 10 days of culture. Although *ex-vivo* responses to the S1 pool were significantly higher than media (**Figure 3B**), responses following cultured ELISpot could not be detected under the same conditions or with double the cell density (10 × 10^3^ cells/well) as CMV-495 (**Figure 3C**). Further increasing cell/well densities were investigated for their capacity to separate S1 recall responses without inducing background visual artefacts. Cell/well densities of 20 × 10^3^ were unable to depict robust recall responses to S1 peptides, whereas 100 × 10^3^ generated background visual artefacts in the chromogenic ELISpot (**Figure 4**). Substituting chromogenic ELISpot for a fluorescent detection modality (Fluorospot) enabled an increased cell density of 100 × 10^3^ cells/well to detect antigen-specific S1 pool IFNγ recall responses without a substantial increase in non-specific background activity (**Figures 3D**, **4G**).

### 3.2 Optimisation of culture period, IL-2 provision, and overnight washing for delineation of cognate central memory antigen recall responses to SARS-CoV-2

**Figure 5:**
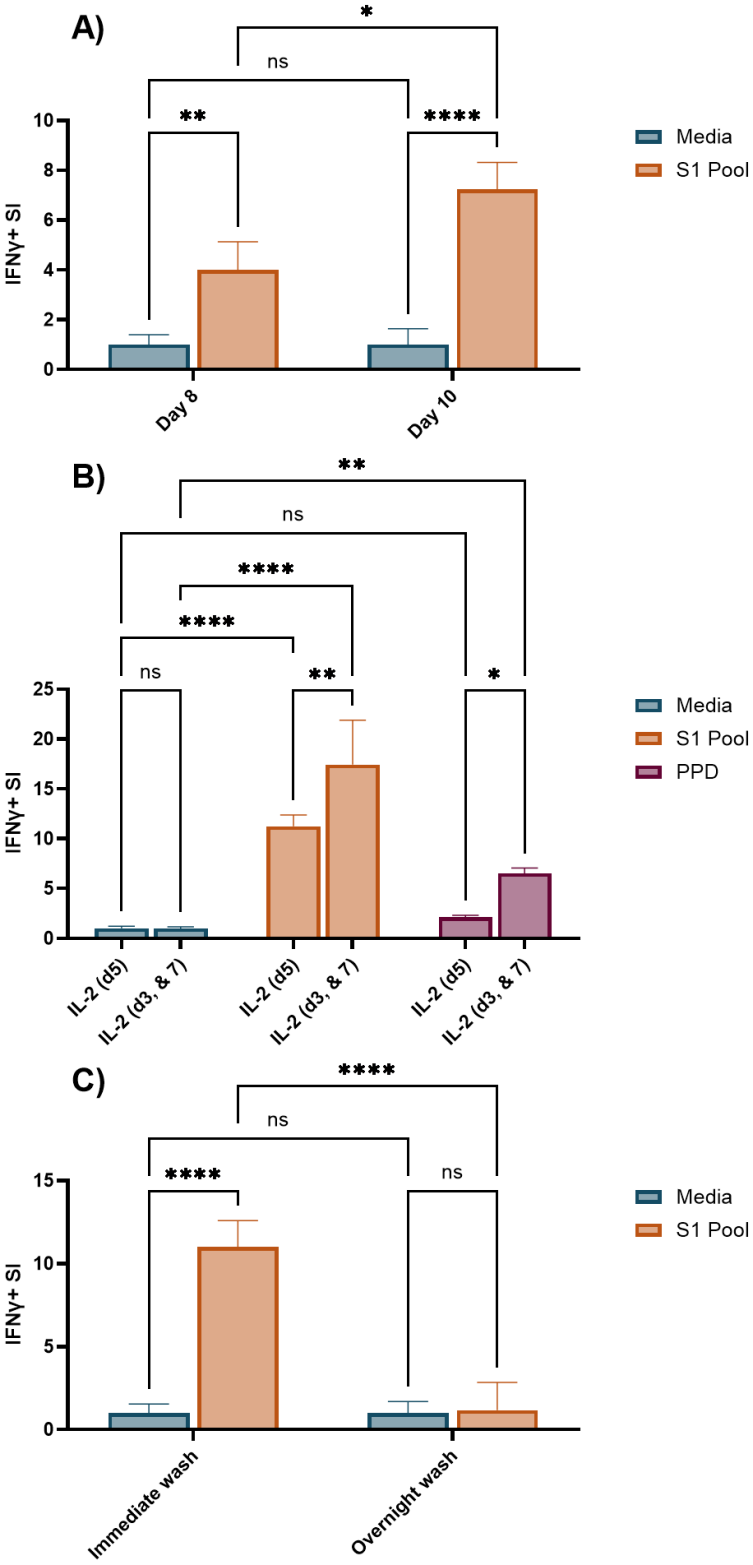
Comparison of an 8- or 10-day culture period, an alternate IL-2 provision schedule, and overnight wash and rest for SARS-CoV-2-primed cultured ELISpot. Unless otherwise stated, all cultured ELISpot assays were primed with the S1 pool and assessed following 10 days of culture with 100 × 10^3^ cells/well in Fluorospot. **(A)** IFNγ stimulation index (SI) to media, and S1 pool following an 8- or 10-day culture. **(B)** IFNγ SI to media, S1 pool, and a non-cognate antigen, purified protein derivative (PPD) with IL-2 supplementation occurring on either day 5 (IL-2 (d5)), or on days 3 and 7 (IL-2 (d3, & 7)) of culture. **(C)** IFNγ SI to media, and S1 pool with cell washes occurring either immediately upon cultured cell collection (immediate wash), or the prior evening with an overnight rest (overnight wash). Graphs present the mean ± standard deviation (SD) of the SI one representative donor [**(A)** donor B, **(B, C)** donor A, **Supplementary Tables S1, S5–S7**] of two–three donors analysed in two to four replicate wells. Two-way ANOVA was used for statistical significance with the following classifications: *p < 0.05, **p < 0.01, ****p < 0.001; ns, not significant.

A significant number of technical variables associated with the cultured ELISpot method have been extensively documented in the literature, highlighting the need for standardised protocols to ensure consistency and reliability in results; key examples are provided in **Table 1**. By incorporating Fluorospot endpoint analysis, our cultured ELISpot protocol achieves a 2.5-fold increase in cell density per well compared with what has been previously documented in the literature (38). As such, we aimed to investigate three of the predominantly altered variables within documented cultured ELISpots for their impact in our Fluorospot analysis of SARS-CoV-2 IFNγ responses. Shortening the culture period from 10 to 8 days reduced recall SI responses of S1 pool primed and re-exposed cultured ELISpot values by 0.45-fold of the 10-day response (**Figure 5A**), indicating a higher assay sensitivity for a 10-day culture. Predominantly, shorter cultured ELISpot protocols of 10 days favours IL-2 provision on days 3 and 7 of culture (6, 7, 23, 38), whereas longer cultures of 14 days delay the initial IL-2 addition to day 5, with a second dose on day 10 (11, 12, 39). During analysis, cognate antigen responses were compared with both media, to define assay sensitivity, and an unrelated recall antigen-purified protein derivative (PPD; an extract from tubercule bacillus, *Mycobacterium tuberculosis*), to define assay specificity. As an unrelated antigen, significantly increased PPD IFNγ responses indicates expansion of non-cognate responses during the cultured ELISpot protocol, detailing a reduced assay specificity for responses to the primed cognate antigen of interest. In 10-day cultures, provision of IL-2 on day 5 of culture demonstrated significant IFNγ recall responses to the cognate S1 pool, without inducing unrelated PPD responses. Meanwhile, an earlier and more frequent provision of IL-2 (days 3 and 7) significantly enhanced both cognate and unrelated PPD responses by 55% and 305%, respectively (**Figure 5B**). Delayed provision of IL-2 on day 5 provided a greater assay specificity, albeit with a slightly reduced cognate antigen sensitivity. Following culture, cells were washed and rested to downregulate pro-inflammatory mechanisms prior to their restimulation during endpoint ELISpot analysis. Published protocols vary washes between overnight with a rest (7, 40) or immediately upon cell collection (11, 12). An overnight wash and rest were not beneficial in our assays, as IFNγ SI was either not impacted (**Supplementary Table S10**) or reduced to 0.11-fold of the recall response observed when cells were washed immediately after collection (**Figure 5C**).

### 3.3 S1 pool-induced IFNγ recall responses are reduced in CCR7-depleted PBMCs

**Figure 6:**
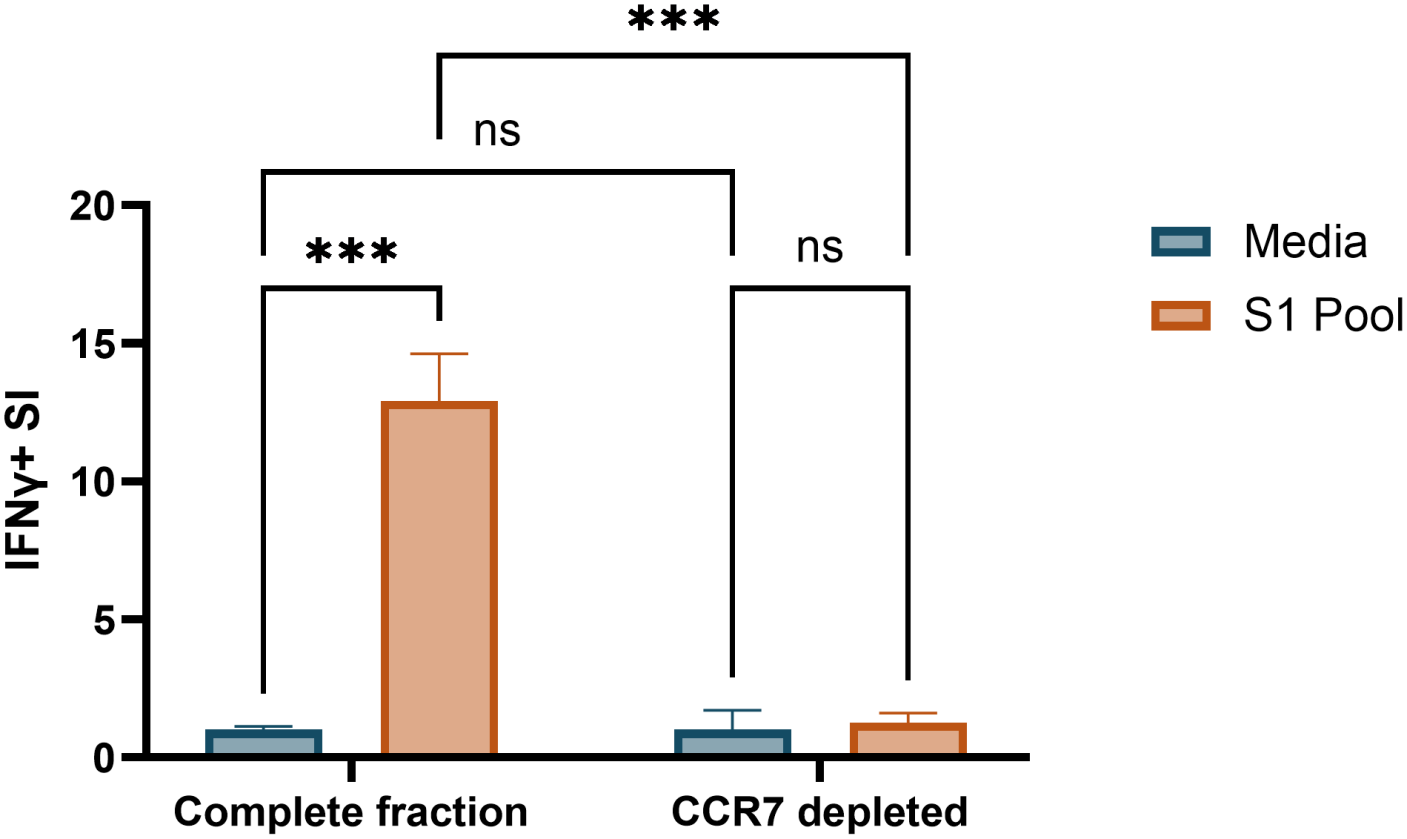
CCR7^+^ cell depletion ablates cultured ELISpot cognate antigen IFNγ responses. IFNγ stimulation index (SI) to media and S1 pool in Fluorospot assay with 100 × 10^3^ cells/well following S1 pool primed 10-day culture in complete and CCR7+ depleted cell fractions. Data are presented as mean ± standard deviation (SD) of duplicate wells from donor A (**Supplementary Table S1**). Two-way ANOVA was used for statistical significance and significance shown as ***p < 0.005; ns, not significant.

Several papers demonstrate cultured ELISpot protocols to reliably detect responses from the central memory T-cell compartment with depletion of CCR7-expressing central memory T cells reducing cognate antigen responses when compared with complete fractions (5–7). Given our modifications to published protocols, we aimed to confirm that we were similarly detecting IFNγ responses from central memory T cells. Depletion of CCR7^+^ PBMCs prior to priming and culture served to selectively remove the central memory T-cell compartment to determine if they were the driving cells of the cultured response. Post-culture viability of CCR7-depleted and complete fraction wells were 72% and 74%, respectively (**Supplementary Table S11**). CCR7 depletion reduced IFNγ SI to 10% of the complete fraction recall response (**Figure 6**), confirming that our cultured ELISpot protocol detects a central memory T-cell driven response, as previously documented in the literature (**Figure 6**) (5–7).

### 3.4 Individual peptide responses including cross-reactive epitopes following pooled peptide-stimulated cultures

**Figure 7:**
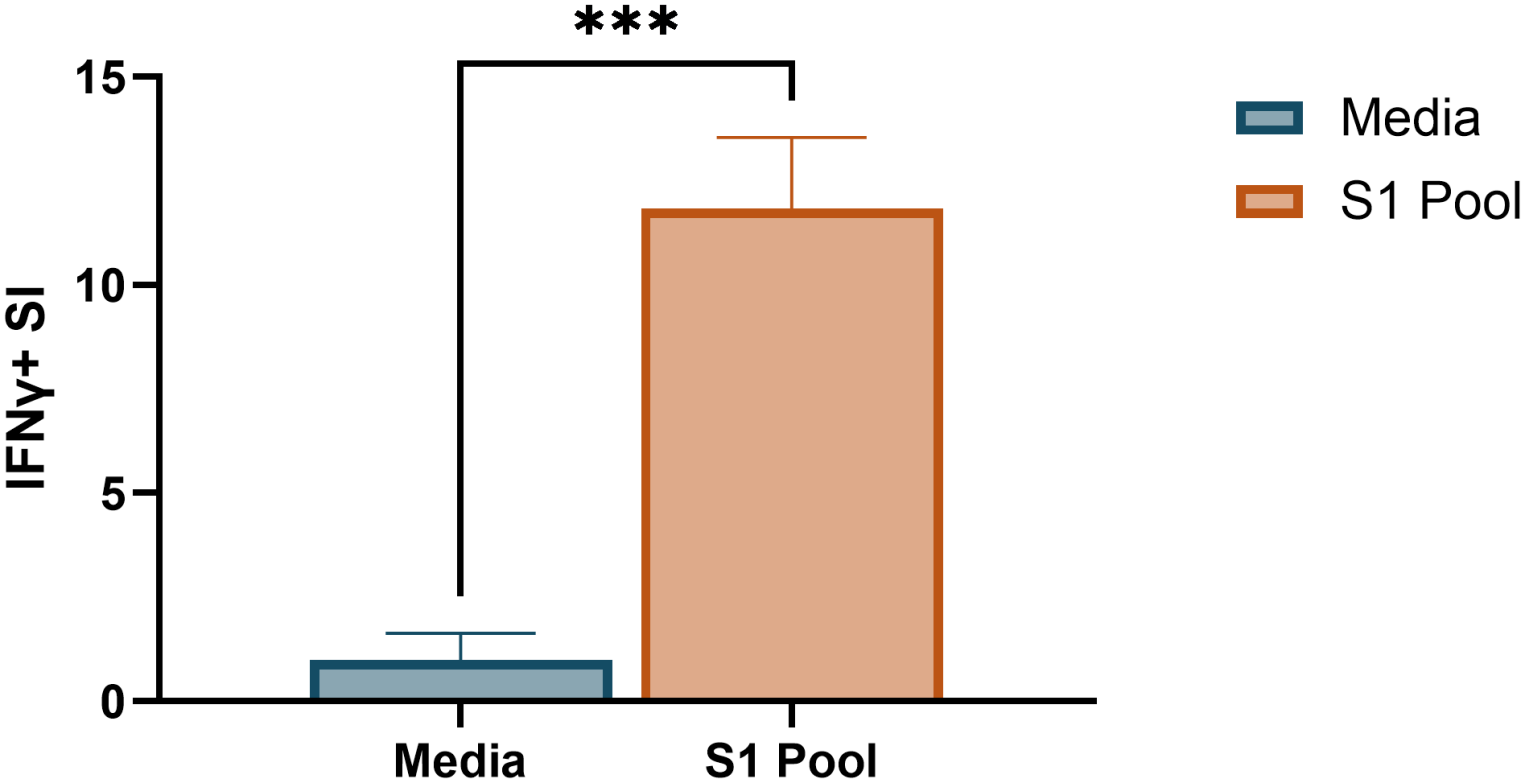
Pooled peptide responses following whole SARS-CoV-2 S-protein-primed cultured ELISpot. Media and S1 pool IFNγ responses in 100 × 10^3^ cells/well Fluorospot following whole SARS-CoV-2 S-protein-primed 10-day cultured ELISpot. Graph depicts the mean ± standard deviation (SD) of the stimulation index (SI) of a representative donor [donor A, (**Supplementary Tables S1**, **S8**] of two donors analysed in two to four replicate wells. Unpaired *t*-test was used for statistical analysis with ***p < 0.005.

**Figure 8:**
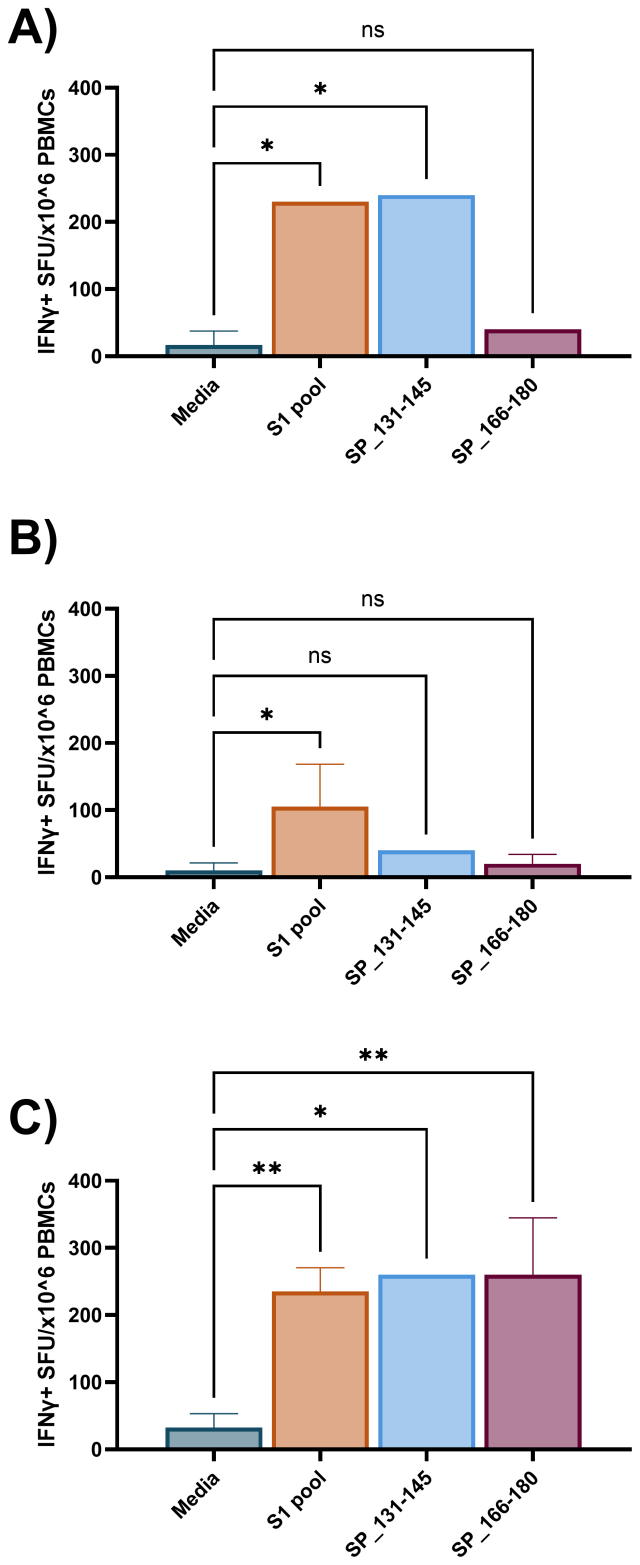
Individual peptide IFNγ recall responses following S1 pool-primed cultured ELISpot. Three individual donor IFNγ recall responses to media, S1 pool, and two individual peptides from the S1 pool (SP_131-145, and the CD4 dominant: SP_166-180) following 10-day cultured ELISpot primed with S1 pool. **(A)** Donor A demonstrated SP_131-145 to be a responding peptide within the S1 pool. **(B)** Donor B responded to neither peptide but responded to the S1 pool and **(C)** donor C recalled to both SP_131-145 and SP_166-180. Graphs present mean ± standard deviation (SD) of the spot forming unit (SFU) per 1 × 10^6^ cells of one to four replicates. One-way ANOVA was used for statistical analysis with * denoting p < 0.05, and **p < 0.01; ns, not significant.

**Figure 9:**
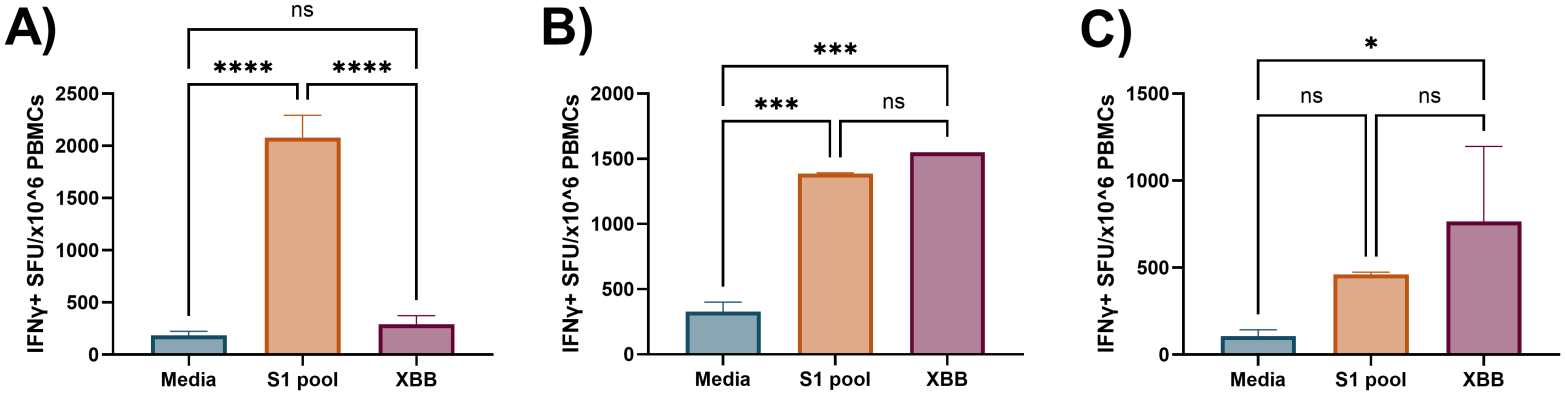
Preliminary demonstration of cross-reactive recall IFNγ responses following S1 peptide pool priming in cultured ELISpot. Three independent donor IFNγ recall responses to media, S1 pool (cognate), and XBB overlapping S1 peptides (XBB, cross-reactive) following cultured ELISpot primed with the SARS-CoV-2 S1 peptide pool. **(A)** Donor A demonstrated no cross-reactive XBB responses following priming culture with the S1 peptide pool. **(B)** Donors C and **(C)** D demonstrated evidence of cross-reactive recall responses to an XBB peptide pool containing XBB.1.5 strain variants of three corresponding peptides to the Wuhan SARS-CoV-2 S1 peptide pool. Graphs are presented as mean ± standard deviation (SD) of spot forming units (SFU) per 1 × 10^6^ cells of one to four replicates. One-way ANOVA was used for statistical analysis with the following classifications: *p < 0.05, ***p < 0.005, ****p < 0.001; ns, not significant.

The requirement for two antigen stimulation periods in cultured ELISpot assays provides the novel capacity to investigate specific responding antigens. Through priming with a broad antigen such as whole protein, and subsequently stimulating with individual peptides during secondary re-exposure in the cultured ELISpot end-point assay, responding peptides and/or cross-reactive epitopes can be delineated. **Figure 7** demonstrates a capacity to recall reactivity to pooled peptides (S1 pool) following *in vitro* priming with whole S-protein. As donor MHC haplotype and environmental exposures may influence individual epitope reactivity, **Figures 8** and **9** show independent data for each donor tested. Indeed, although all three donors had an IFNγ response to S1 pool recall, responses to two of the individual peptides present within the pool varied. Donor A positively recalled to SP_131-145 (**Figure 8A**), donor B to neither peptide (**Figure 8B**), and donor C to both SP_131-145 and SP_166-180 independently (**Figure 8C**). Furthermore, we investigated whether our assay could be used to examine cross-reactive recall responses to variant peptides, through initial culture priming with reference antigens such as our S1 pool, and subsequent restimulation during end-point analysis with variant peptides such as from XBB.1.5 SARS-CoV-2. Positive recall responses are indicative of potential cross-reactivity, either from preexisting central memory XBB-reactive T cells receiving sufficient survival signals during reference antigen priming, or from reference-primed central memory T cells demonstrating capacity for recall responses to XBB.1.5 variant peptides. Following culture primed with the S1 pool, we demonstrate variable capacities for the induction of potential cross-reactive IFNγ recall responses to a pool of three S1 overlapping XBB.1.5 viral variant peptides. Two out of the three donors demonstrated a significant cross-reactive response when primed with S1 pool and restimulated with the XBB variant pool when compared with media. Indeed, donor A demonstrated a non-cross-reactive recall response despite significant IFNγ release following S1 pool restimulation (**Figure 9A**), whereas donors B and D showed evidence for cross-reactive IFNγ recall responses with significant IFNγ release following XBB.1.5 variant peptide restimulation as compared with media. Of particular interest was no significant change in the magnitude of IFNγ SFU between S1 pool and XBB.1.5 peptide restimulation for donors B and D (**Figures 9B, C**).

## 4 Discussion

Cultured ELISpot protocol design requires consideration of the culture length, survival signal provision (IL-2), and endpoint analysis modality (chromogenic vs. Fluorospot) to facilitate sensitive and antigen-specific recall of central memory T-cell responses. While many of the published protocols will generate measurable results to a varying extent, our study showed weaker immune responses to require a more robust protocol, requiring at least 10 days in culture and delayed provision of growth factors (IL-2 on day 5) (**Figure 5**). Although both chromogenic ELISpot and Fluorospot require similar stages of initial analyte capture during an incubation process and then antibody sandwich detection of the captured analytes, their method of detection differs. Chromogenic ELISpot utilises colorimetric detection such as streptavidin–alkaline phosphatase to generate a colorimetric stain through reaction with chromogenic substances such as a colorimetric AP kit, whereas Fluorospot directly tags the secondary detection antibody with one of four fluorescent markers for excitation/detection at wavelengths of 380nm/430nm, 490nm/510nm, 550nm/570nm, and 640nm/660nm, providing the capacity for multiplex detection of co-expressed cytokines from individual cells and increased sensitivity as compared with flow cytometry (21). Certainly, our analysis demonstrates an enhanced sensitivity for the Fluorospot technique with a higher than previously reported input of cultured cells (2.5-fold higher) over chromogenic ELISpot for the post-culture quantification of central memory T cells, to enable the determination of antigen–cell interactions for lower frequency T cells, such as those to some SARS-CoV-2 epitopes (**Figures 3**, **4**).

Specifically, we observed that an extension of the culture period from 8 to 10 days led to a substantial increase in SI (**Figure 5A**), indicating enhanced assay sensitivity with the longer culture period, potentially due to an enhanced proliferative window for cognate central memory T cells (8). The cultured ELISpot protocol treads a fine line between providing survival signals to enhance central memory T-cell priming, without enhancing the survival of non-specific, non-central memory T cells capable of IFNγ production, such as effector T cells (41) or innate immune cells such as natural killer (NK) cells (42). Within published cultured ELISpot protocols, shorter culture periods are associated with earlier provision of IL-2 (6, 38). Within our system, an earlier schedule for provision of IL-2 (day 3 and day 7) enhanced both the cognate S1 pool and unrelated PPD recall responses, whereas IL-2 provision on day 5 supported the specific detection of responses to the priming antigen (**Figure 5B**). Given the stringent requirement for specificity for cognate–antigen responses when investigating individual and cross-reactive peptide reactivity, we concluded that the slight reduction in cognate antigen sensitivity to be a worthwhile compromise for an enhanced assay specificity using the delayed IL-2 protocol. Further optimisations of survival signal provision for cultured ELISpots may also include the provision of non-IL-2 survival signals such as IL-7 and IL-15 (40, 43). Cell washing is critical to enhancing endpoint assay sensitivity for cultured ELISpot, as it removes potentially pro-inflammatory cytokines within the cell media capable of enhancing non-specific antigen responses upon endpoint ELISpot analysis. We demonstrated a preference for the cell wash to occur immediately after culture cell collection rather than the evening before with an overnight rest, as the latter reduced IFNγ SI (**Figure 5C**). Multiple studies have demonstrated the specificity for central memory T-cell responses in the cultured ELISpot assay via CCR7^+^ cell depletion (5–7). Our results further replicated these studies, confirming our assay conditions to similarly enable the study of central memory T cells (**Figure 6**).

We further explored the assay’s capacity for 1) recalling responses to pooled peptide epitopes following whole protein antigen priming, 2) independent single-peptide epitope responses following pooled peptide priming, 3) and cross-reactivity with viral variant peptides. The initial results support the potential flexibility that this assay may offer for the study of central memory T-cell responses. Contrasting the single provision of recall antigen during *ex-vivo* ELISpot, the requirement for a priming followed by a recall antigen in the cultured ELISpot technique provides a unique capacity to delineate individual antigenic peptides driving whole protein or peptide pool responses (23). When investigating individualised responses, such as to responding peptides, the donor’s past exposure to antigen (44) or HLA haplotype (45, 46) can significantly vary results. As observed in **Figure 8**, despite all three donors responding to the S1 peptide pool, each demonstrated varied capacity for IFNγ release following individual recall of S1 peptide. This demonstrates that our cultured ELISpot protocol can delineate individually responding peptides following peptide pool-primed culture. Additionally, when whole S-protein was used for initial stimulation, subsequent pooled peptide responses were observed, suggesting a potential capacity for our assay to delineate the individual responding peptides following whole protein priming (23) (**Figure 7**). Furthering the concept of delineating individual peptide responses, we next examined the potential for our assay to investigate cross-reactive cellular immune responses. Following culture primed with the S1 peptide pool, we examined recall responses to the identical S1 pool, and a pool of three overlapping SARS-CoV-2 XBB.1.5 strain variant peptides, demonstrating functional recall responses in two of three donors assessed (**Figure 9**). Given the numerous and ever evolving collection of SARS-CoV-2 variants following the pandemic (47, 48), vaccine formulations need to remain competitive with current variant strains to offer beneficial populational protective immunity (49). As such, regions containing various viral variants should be investigated not only for altered immunogenicity but for potential protective cross-reactivity. The capacity of our cultured ELISpot protocol to demonstrate cross-reactive recall responses at an individualised donor level may provide an assistive tool for the literature for future investigations for beneficial cross-reactive immunogenic sites to facilitate vaccine development capable of expanding either the breadth or longevity of protection conferred (50, 51). Furthermore, the variable cross-reactive responses observed between the donors investigated in **Figure 9** stresses the importance for subsequent studies aimed at specifically describing cross-reactive peptides to consider not only an individual’s HLA haplotype but also those within the population of interest to ensure identified cross-reactive sequences are beneficial across the populational level (49, 52–54).

Our study aimed to investigate the potential for our cultured ELISpot protocol to delineate individual or cross-reactive peptide responses but is limited by the few donors examined to conclusively identify the individual peptides driving recall responses to the S1 pool. For future studies implementing our optimised cultured ELISpot protocol, we recommend users to consider stratifying donors based on *ex-vivo* antigen responsiveness and HLA haplotype to ensure a robust delineation of individual peptides driving pooled responses, or the capacity for cross-reactivity on not only an individualised, but a populational stratification. The capacity to identify individual peptide responses, along with the demonstration of cross-reactivity between viral variants, may offer a valuable approach for investigating how viral variants influence the functional central memory T-cell response. This combined capacity can enhance our understanding of immune system adaptability and effectiveness in the face of evolving viral challenges, ultimately aiding in the development of more effective vaccines and therapeutic strategies.

## Data Availability

The original contributions presented in the study are included in the article/**Supplementary Material**. Further inquiries can be directed to the corresponding author.

## References

[1] Farber DL Yudanin NA Restifo NP. Human memory T cells: generation, compartmentalization and homeostasis. Nat Rev Immunol. (2014) 14:24–35. doi: 10.1038/nri3567 24336101 PMC4032067

[2] Miller JD van der Most RG Akondy RS Glidewell JT Albott S Masopust D. Human effector and memory CD8+ T cell responses to smallpox and yellow fever vaccines. Immunity. (2008) 28:710–22. doi: 10.1016/j.immuni.2008.02.020 18468462

[3] Gray JI Westerhof LM MacLeod MKL. The roles of resident, central and effector memory CD4 T-cells in protective immunity following infection or vaccination. Immunology. (2018) 154:574–81. doi: 10.1111/imm.12929 PMC6050220 29570776

[4] Masopust D Schenkel JM. The integration of T cell migration, differentiation and function. Nat Rev Immunol. (2013) 13:309–20. doi: 10.1038/nri3442 23598650

[5] Godkin AJ Thomas HC Openshaw PJ. Evolution of epitope-specific memory CD4(+) T cells after clearance of hepatitis C virus. J Immunol. (2002) 169:2210–4. doi: 10.4049/jimmunol.169.4.2210 12165552

[6] Calarota SA Baldanti F. Enumeration and characterization of human memory T cells by enzyme-linked immunospot assays. Clin Dev Immunol. (2013) 2013:637649. doi: 10.1155/2013/637649 24319467 PMC3844203

[7] Todryk SM Pathan AA Keating S Porter DW Berthoud T Thompson F. The relationship between human effector and memory T cells measured by ex vivo and cultured ELISPOT following recent and distal priming. Immunology. (2009) 128:83–91. doi: 10.1111/j.1365-2567.2009.03073.x 19689738 PMC2747141

[8] Whitmire JK Eam-B-Fau-Whitton JL Whitton JL. Tentative T cells: memory cells are quick to respond, but slow to divide. PLoS Pathway (2008) 4(4), 1553–7374. doi: 10.1371/journal.ppat.1000041 PMC2275797 18404208

[9] Janeway CA Jr Travers P Walport M Shlomchik MJ. Immunobiology: The Immune System in Health and Disease. 5th edition. New York: Garland Science (2001).

[10] Riddell NE. Immune responses: primary and secondary. eLS (2020), 316–26. doi: 10.1002/9780470015902.a0029196

[11] Flanagan KL Lee EA Gravenor MB Reece WH Urban BC Doherty T. Unique T cell effector functions elicited by Plasmodium falciparum epitopes in malaria-exposed Africans tested by three T cell assays. J Immunol. (2001) 167:4729–37. doi: 10.4049/jimmunol.167.8.4729 11591804

[12] Reece WH Pinder M Gothard PK Milligan P Bojang K Doherty T. A CD4(+) T-cell immune response to a conserved epitope in the circumsporozoite protein correlates with protection from natural Plasmodium falciparum infection and disease. Nat Med. (2004) 10:406–10. doi: 10.1038/nm1009 15034567

[13] Huang Y Yang C Xu XF Xu W Liu SW. Structural and functional properties of SARS-CoV-2 spike protein: potential antivirus drug development for COVID-19. Acta Pharmacol Sin. (2020) 41:1141–9. doi: 10.1038/s41401-020-0485-4 PMC7396720 32747721

[14] Dai L Gao GF. Viral targets for vaccines against COVID-19. Nat Rev Immunol. (2021) 21:73–82. doi: 10.1038/s41577-020-00480-0 33340022 PMC7747004

[15] Cremoni M Allouche J Graca D Zorzi K Fernandez C Teisseyre M. Low baseline IFN-gamma response could predict hospitalization in COVID-19 patients. Front Immunol. (2022) 13:953502. doi: 10.3389/fimmu.2022.953502 36225915 PMC9548596

[16] Foster WS Lee JL Thakur N Newman J Spencer AJ Davies S. Tfh cells and the germinal center are required for memory B cell formation & humoral immunity after ChAdOx1 nCoV-19 vaccination. Cell Rep Med. (2022) 3:100845. doi: 10.1016/j.xcrm.2022.100845 36455555 PMC9663747

[17] Schwarzkopf S Krawczyk A Knop D Klump H Heinold A Heinemann FM. Cellular immunity in COVID-19 convalescents with PCR-confirmed infection but with undetectable SARS-coV-2–specific igG. Emerging Infect Diseases. (2021) 27:122–9. doi: 10.3201/2701.203772 33058753

[18] Lin H Zhang J Dong S Liu Y Liu P Gao GF. An adjusted ELISpot-based immunoassay for evaluation of SARS-CoV-2-specific T-cell responses. Biosaf Health. (2022) 4:179–85. doi: 10.1016/j.bsheal.2022.04.005 PMC9047432 35505811

[19] Rumke LW Smit WL Bossink A Limonard GJM Muilwijk D Haas LEM. Impaired SARS-CoV-2 specific T-cell response in patients with severe COVID-19. Front Immunol. (2023) 14:1046639. doi: 10.3389/fimmu.2023.1046639 37168853 PMC10165493

[20] Braudeau C Salabert-Le-Guen N Chevreuil J Rimbert M Martin JC Josien R. An easy and reliable whole blood freezing method for flow cytometry immuno-phenotyping and functional analyses. Cytometry Part B: Clin Cytometry. (2021) 100:652–65. doi: 10.1002/cyto.b.v100.6 33544978

[21] Chauvat A Benhamouda N Gey A Lemoine FM Paulie S Carrat F. Clinical validation of IFNγ/IL-10 and IFNγ/IL-2 FluoroSpot assays for the detection of Tr1 T cells and influenza vaccine monitoring in humans. Hum Vaccines Immunotherapeutics. (2013) 10:104–13. doi: 10.4161/hv.26593 PMC4181035 24084262

[22] Malyguine A Strobl SL Shafer-Weaver KA Ulderich T Troke A Baseler M. A modified human ELISPOT assay to detect specific responses to primary tumor cell targets. J Transl Med (2004) 2(1), 1479–5876. doi: 10.1186/1479-5876-2-9 PMC415560 15050026

[23] Jeewandara C Ogg GS Malavige GN. Cultured ELISpot Assay to Investigate Dengue Virus Specific T-Cell Responses. In: Kalyuzhny AE, editor. Handbook of ELISPOT: Methods and Protocols. Springer New York, New York, NY (2018). p. 165–71. 10.1007/978-1-4939-8567-8_14 29956182

[24] Harris PA Taylor R Thielke R Payne J Gonzalez N Conde JG. Research electronic data capture (REDCap)—A metadata-driven methodology and workflow process for providing translational research informatics support. J Biomed Informatics. (2009) 42:377–81. doi: 10.1016/j.jbi.2008.08.010 PMC2700030 18929686

[25] Harris PA Taylor R Minor BL Elliott V Fernandez M O'Neal L. The REDCap consortium: Building an international community of software platform partners. J Biomed Inf. (2019) 95. doi: 10.1016/j.jbi.2019.103208 PMC7254481 31078660

[26] Tools IED. (2022). Available online at: https://www.iedb.org/ (Accessed August 1, 2023).

[27] Gonzalez-Galarza FF McCabe A Santos E Jones J Takeshita L Ortega-Rivera ND. Allele frequency net database (AFND) 2020 update: gold-standard data classification, open access genotype data and new query tools. Nucleic Acids Res. (2020) 48:D783–D8. doi: 10.1093/nar/gkz1029 PMC7145554 31722398

[28] Tye EXC Jinks E Haigh TA Kaul B Patel P Parry HM. Mutations in SARS-CoV-2 spike protein impair epitope-specific CD4(+) T cell recognition. Nat Immunol. (2022) 23:1726–34. doi: 10.1038/s41590-022-01351-7 36456735

[29] GISAID. (2023). Available online at: https://gisaid.org/hcov19-mutation-dashboard/ (Accessed July 20, 2023).

[30] Zaia JA Gallez-Hawkins G Li X Yao ZQ Lomeli N Molinder K. Infrequent occurrence of natural mutations in the pp65(495-503) epitope sequence presented by the HLA A*0201 allele among human cytomegalovirus isolates. J Virol. (2001) 75:2472–4. doi: 10.1128/JVI.75.5.2472-2474.2001 PMC114832 11160752

[31] Flanagan KL Mwangi T Plebanski M Odhiambo K Ross A Sheu E. Ex vivo interferon-gamma immune response to thrombospondin-related adhesive protein in coastal Kenyans: longevity and risk of Plasmodium falciparum infection. Am J Trop Med Hyg (2003) 68(4), 0002–9637. doi: 10.4269/ajtmh.2003.68.421 12875291

[32] Xiang SD Gao Q Wilson KL Heyerick A Plebanski M. Mapping T and B cell epitopes in sperm protein 17 to support the development of an ovarian cancer vaccine. Vaccine. (2015) 33:5950–9. doi: 10.1016/j.vaccine.2015.07.094 26263201

[33] Wilson KL Pouniotis D Hanley J Xiang SD Ma C Coppel RL. A synthetic nanoparticle based vaccine approach targeting MSP4/5 is immunogenic and induces moderate protection against murine blood-stage malaria. Front Immunol. (2019) 10:331. doi: 10.3389/fimmu.2019.00331 30930890 PMC6428706

[34] Todryk SM Bejon P Mwangi T Plebanski M Urban B Marsh K. Correlation of memory T cell responses against TRAP with protection from clinical malaria, and CD4 CD25 high T cells with susceptibility in Kenyans. PloS One. (2008) 3:e2027. doi: 10.1371/journal.pone.0002027 18446217 PMC2323567

[35] Hasan AN Doubrovina E Sottile R Prockop S Klatt MG Heller G. Dominant epitopes presented by prevalent HLA alleles permit wide use of banked CMVpp65 T cells in adoptive therapy. Blood Adv. (2022) 6:4859–72. doi: 10.1182/bloodadvances.2022007005 PMC9631666 35605246

[36] Ohnishi M Sakurai T Heike Y Yamazaki R Kanda Y Takaue Y. Evaluation of cytomegalovirus-specific T-cell reconstitution in patients after various allogeneic haematopoietic stem cell transplantation using interferon-gamma-enzyme-linked immunospot and human leucocyte antigen tetramer assays with an immunodominant T-cell epitope. Br J Haematol. (2005) 131:472–9. doi: 10.1111/j.1365-2141.2005.05800.x 16281937

[37] Seale H MacIntyre CR Gidding HF Backhouse JL Dwyer DE Gilbert L. National serosurvey of cytomegalovirus in Australia. Clin Vaccine Immunol. (2006) 13:1181–4. doi: 10.1128/CVI.00203-06 PMC1656547 16957061

[38] Keating SM Bejon P Berthoud T Vuola JM Todryk S Webster DP. Durable human memory T cells quantifiable by cultured enzyme-linked immunospot assays are induced by heterologous prime boost immunization and correlate with protection against malaria. J Immunol. (2005) 175:5675–80. doi: 10.4049/jimmunol.175.9.5675 16237057

[39] Pinder M Reece WH Plebanski M Akinwunmi P Flanagan KL Lee EA. Cellular immunity induced by the recombinant Plasmodium falciparum malaria vaccine, RTS,S/AS02, in semi-immune adults in The Gambia. Clin Exp Immunol. (2004) 135:286–93. doi: 10.1111/j.1365-2249.2004.02371.x PMC1808944 14738458

[40] Chen C Jiang X Liu X Guo L Wang W Gu S. Identification of the association between HBcAg-specific T cell and viral control in chronic HBV infection using a cultured ELISPOT assay. J Leukoc Biol. (2021) 109:455–65. doi: 10.1002/JLB.5MA0620-023RR 32620046

[41] Ross SH Cantrell DA. Signaling and function of interleukin-2 in T lymphocytes. Annu Rev Immunol. (2018) 36:411–33. doi: 10.1146/annurev-immunol-042617-053352 PMC6472684 29677473

[42] Wang KS Frank DA Ritz J. Interleukin-2 enhances the response of natural killer cells to interleukin-12 through up-regulation of the interleukin-12 receptor and STAT4. Blood. (2000) 95:3183–90. doi: 10.1182/blood.V95.10.3183 10807786

[43] Jennes W Kestens L Nixon DF Shacklett BL. Enhanced ELISPOT detection of antigen-specific T cell responses from cryopreserved specimens with addition of both IL-7 and IL-15—the Amplispot assay. J Immunol Methods. (2002) 270:99–108. doi: 10.1016/S0022-1759(02)00275-2 12379342

[44] Simhadri VL Hopkins L McGill JR Duke BR Mukherjee S Zhang K. Cas9-derived peptides presented by MHC Class II that elicit proliferation of CD4(+) T-cells. Nat Commun. (2021) 12:5090. doi: 10.1038/s41467-021-25414-9 34429421 PMC8384835

[45] Karnaukhov V Paes W Woodhouse IB Partridge T Nicastri A Brackenridge S. HLA variants have different preferences to present proteins with specific molecular functions which are complemented in frequent haplotypes. Front Immunol. (2022) 13:1067463. doi: 10.3389/fimmu.2022.1067463 36605212 PMC9808399

[46] James EA Moustakas AK Bui J Nouv R Papadopoulos GK Kwok WW. The binding of antigenic peptides to HLA-DR is influenced by interactions between pocket 6 and pocket 9. J Immunol. (2009) 183:3249–58. doi: 10.4049/jimmunol.0802228 PMC4061985 19648278

[47] Zabidi NZ Liew HL Farouk IA Puniyamurti A Yip AJW Wijesinghe VN. Evolution of SARS-coV-2 variants: implications on immune escape, vaccination, therapeutic and diagnostic strategies. Viruses. (2023) 15, 3–7. doi: 10.3390/v15040944 PMC10145020 37112923

[48] Harvey WT Carabelli AM Jackson B Gupta RK Thomson EC Harrison EM. SARS-CoV-2 variants, spike mutations and immune escape. Nat Rev Microbiol. (2021) 19:409–24. doi: 10.1038/s41579-021-00573-0 PMC8167834 34075212

[49] Lugano D Kutima B Kimani M Sigilai A Gitonga J Karani A. Evaluation of population immunity against SARS-CoV-2 variants, EG.5.1, FY.4, BA.2.86, JN.1, JN.1.4, and KP.3.1.1 using samples from two health demographic surveillance systems in Kenya. BMC Infect Dis. (2024) 24:1474. doi: 10.1186/s12879-024-10367-3 39732637 PMC11682625

[50] Vojtek I Buchy P Doherty TM Hoet B. Would immunization be the same without cross-reactivity? Vaccine. (2019) 37:539–49. doi: 10.1016/j.vaccine.2018.12.005 30591255

[51] Vinzon SE Lopez MV Cafferata EGA Soto AS Berguer PM Vazquez L. Cross-protection and cross-neutralization capacity of ancestral and VOC-matched SARS-CoV-2 adenoviral vector-based vaccines. NPJ Vaccines. (2023) 8:149. doi: 10.1038/s41541-023-00737-4 37794010 PMC10550992

[52] Kennedy RB Ovsyannikova IG Lambert ND Haralambieva IH Poland GA. The personal touch: strategies toward personalized vaccines and predicting immune responses to them. Expert Rev Vaccines. (2014) 13:657–69. doi: 10.1586/14760584.2014.905744 PMC4073206 24702429

[53] McCallum MA-O Czudnochowski NA-O Rosen LA-O Zepeda SA-O Bowen JA-O Walls AA-O. Structural basis of SARS-CoV-2 Omicron immune evasion and receptor engagement. Science. (2022) 375(6583), 1095–9203. doi: 10.1101/2021.12.28.474380 PMC9427005 35076256

[54] Furnon W Cowton VM De Lorenzo G Orton R Herder V Cantoni D. Phenotypic evolution of SARS-CoV-2 spike during the COVID-19 pandemic. Nat Microbiol. (2025) 10:77–93. doi: 10.1038/s41564-024-01878-5 39753670 PMC11726466

[55] Jerome J Plebanski M. (2024). Available online at: https://BioRender.com/t03z969 (Accessed November 28, 2024).

[56] Jerome J Plebanski M. (2024). Available online at: https://BioRender.com/g93u848 (Accessed November 28, 2024).

