# Supplementary material for: Optimisation of the cultured ELISpot/Fluorospot technique for the selective investigation of SARS-CoV-2 reactive central memory T cells
Source: Front Immunol. 2025 Apr 15;16:1547220. doi: 10.3389/fimmu.2025.1547220 (PMC12037488; doi:10.3389/fimmu.2025.1547220)
Supplement: Supplementary file 1 [file DataSheet1.docx]

# Supplementary data

**Table S1. Donor sex and age**

| Donor Label | Sex | Age |
| --- | --- | --- |
| Donor A | Female | na |
| Donor B | Female | 26 |
| Donor C | Male | 36 |
| Donor D | Male | 57 |
| Donor E | Male | 58 |
| Donor F | Female | na |
| Donor G | Female | 65 |

** na indicates unavailable information*

**Table S2. Individual donor stimulation index IFNγ responses for Figure 3A and B**

| Figure | Donor ID | Condition | Stimulation index (SI) as compared to medias |
| --- | --- | --- | --- |
| Figure 3A | *Donor A | Media (CMV-495) | 1.00 |
|  |  | Media (CMV-495) | 1.00 |
|  |  | CMV-495 | 121.00 |
|  |  | CMV-495 | 100.00 |
|  | Donor F | Media | 1.00 |
|  |  | Media | 1.00 |
|  |  | CMV-495 | 62.50 |
|  |  | CMV-495 | 54.00 |
| Figure 3B | *Donor A | Media (S1) | 1.50 |
|  |  | Media + NaOH (S1) | 0.50 |
|  |  | Media + DMSO (S1) | 1.00 |
|  |  | S1 | 7.50 |
|  |  | S1 | 7.50 |
|  | Donor G | Media | 1.13 |
|  |  | Media + NaOH | 0.75 |
|  |  | Media + DMSO | 1.13 |
|  |  | S1 | 7.13 |
|  |  | S1 | 6.00 |

*All conditions plated at 350K cells per well*

** Indicates representative donor presented in figure*

**Table S3. Individual donor stimulation index IFNγ responses for Figure 3C**

| Figure | Donor ID | Condition | Cell/Well | Stimulation index (SI) as compared to medias |
| --- | --- | --- | --- | --- |
| Figure 3C | *Donor A | Media (CMV-495) | 5K | 0.56 |
|  |  | Media (CMV-495) | 5K | 1.44 |
|  |  | CMV-495 | 5K | 10.06 |
|  |  | CMV-495 | 5K | 9.72 |
|  |  | Media (S1) | 5K | 0.38 |
|  |  | Media + NaOH (S1) | 5K | 0.88 |
|  |  | Media + DMSO (S1) | 5K | 1.75 |
|  |  | S1 | 5K | 0.75 |
|  |  | S1 | 5K | 1.25 |
|  | Donor E | Media | 5K | 0.00 |
|  |  | Media | 5K | 2.00 |
|  |  | CMV-495 | 5K | 277.00 |
|  |  | CMV-495 | 5K | 285.00 |
|  | Donor B | Media (S1) | 10K | 1.33 |
|  |  | Media (S1) | 10K | 1.02 |
|  |  | Media + NaOH (S1) | 10K | 1.02 |
|  |  | Media + NaOH (S1) | 10K | 0.85 |
|  |  | Media + DMSO (S1) | 10K | 0.48 |
|  |  | Media + DMSO (S1) | 10K | 1.30 |
|  |  | S1 | 10K | 0.92 |
|  |  | S1 | 10K | 1.33 |

** Indicates representative donor presented in figure*

**Table S4. Individual donor stimulation index IFNγ responses for Figure 3D**

| Figure | Donor ID | Condition | Stimulation index (SI) as compared to medias |
| --- | --- | --- | --- |
| Figure 3D | *Donor A | Media | 0.97 |
|  |  | Media | 0.81 |
|  |  | Media + NaOH | 0.92 |
|  |  | Media + DMSO | 1.30 |
|  |  | S1 | 12.59 |
|  |  | S1 | 10.43 |
|  | Donor B | Media | 1.54 |
|  |  | Media | 1.54 |
|  |  | Media + NaOH | 0.31 |
|  |  | Media + DMSO | 0.62 |
|  |  | S1 | 8.00 |
|  |  | S1 | 6.46 |
|  | Donor C | Media | 0.00 |
|  |  | Media | 2.00 |
|  |  | Media + NaOH | 0.00 |
|  |  | Media + DMSO | 2.00 |
|  |  | S1 | 15.00 |
|  |  | S1 | 6.00 |

*All conditions were plated at 100K cells per well*

** Indicates representative donor presented in figure*

**Table S5. Individual donor stimulation index IFNγ responses for Figure 5A**

| Figure | Donor ID | Condition | Stimulation index (SI) as compared to medias |
| --- | --- | --- | --- |
| Figure 5A | *Donor B | Media (d8) | 1.20 |
|  |  | Media (d8) | 0.40 |
|  |  | Media + NaOH (d8) | 1.20 |
|  |  | Media + DMSO (d8) | 1.20 |
|  |  | S1 (d8) | 3.20 |
|  |  | S1 (d8) | 4.80 |
|  |  | Media (d10) | 1.54 |
|  |  | Media (d10) | 1.54 |
|  |  | Media + NaOH (d10) | 0.31 |
|  |  | Media + DMSO (d10) | 0.62 |
|  |  | S1 (d10) | 8.00 |
|  |  | S1 (d10) | 6.46 |
|  | Donor C | Media (8) | 1.13 |
|  |  | Media + NaOH (d8) | 1.50 |
|  |  | Media + DMSO (d8) | 0.38 |
|  |  | S1 (d8) | 2.63 |
|  |  | S1 (d8) | 2.63 |
|  |  | Media (d10) | 0.00 |
|  |  | Media (d10) | 2.00 |
|  |  | Media + NaOH (d10) | 0.00 |
|  |  | Media + DMSO (d10) | 2.00 |
|  |  | S1 (d10) | 15.00 |
|  |  | S1 (d10) | 6.00 |
|  | Donor A | Media (8) | 1.00 |
|  |  | Media + NaOH (d8) | 2.00 |
|  |  | Media + DMSO (d8) | 0.00 |
|  |  | S1 (d8) | 5.00 |
|  |  | Media (d10) | 0.00 |
|  |  | Media + NaOH (d10) | 2.40 |
|  |  | Media + DMSO (d10) | 0.60 |
|  |  | S1 (d10) | 13.80 |

*All conditions were plated at 100K cells per well*

** Indicates representative donor presented in figure*

**Table S6. Individual donor stimulation index IFNγ responses for Figure 5B**

| Figure | Donor ID | Condition | Stimulation index (SI) as compared to medias |
| --- | --- | --- | --- |
| Figure 5B | *Donor A | Media (Control) | 0.97 |
|  |  | Media (Control) | 0.81 |
|  |  | Media + NaOH (Control) | 0.92 |
|  |  | Media + DMSO (Control) | 1.30 |
|  |  | S1 (Control) | 12.59 |
|  |  | S1 (Control) | 10.43 |
|  |  | PPD (Control) | 2.00 |
|  |  | PPD (Control) | 2.27 |
|  |  | Media (Alt-IL-2) | 0.81 |
|  |  | Media (Alt-IL-2) | 0.93 |
|  |  | Media + NaOH (Alt-IL-2) | 1.10 |
|  |  | Media + DMSO (Alt-IL-2) | 1.16 |
|  |  | S1 (Alt-IL-2) | 14.26 |
|  |  | S1 (Alt-IL-2) | 20.58 |
|  |  | PPD (Alt-IL-2) | 6.90 |
|  |  | PPD (Alt-IL-2) | 6.14 |
|  | Donor D | Media (Control) | 0.67 |
|  |  | Media (Control) | 0.86 |
|  |  | Media + NaOH (Control) | 1.52 |
|  |  | Media + DMSO (Control) | 0.95 |
|  |  | S1 (Control) | 4.48 |
|  |  | S1 (Control) | 4.29 |
|  |  | PPD (Control) | 2.29 |
|  |  | PPD (Control) | 1.33 |
|  |  | Media (Alt-IL-2) | 1.00 |
|  |  | Media (Alt-IL-2) | 0.17 |
|  |  | Media + NaOH (Alt-IL-2) | 0.67 |
|  |  | Media + DMSO (Alt-IL-2) | 2.17 |
|  |  | S1 (Alt-IL-2) | 8.67 |
|  |  | S1 (Alt-IL-2) | 6.83 |
|  |  | PPD (Alt-IL-2) | 3.33 |
|  |  | PPD (Alt-IL-2) | 1.17 |

*All conditions were plated at 100K cells per well*

** Indicates representative donor presented in figure*

**Table S7. Individual donor stimulation index IFNγ responses for Figure 5C**

| Figure | Donor ID | Condition | Stimulation index (SI) as compared to medias |
| --- | --- | --- | --- |
| Figure 5C | *Donor A | Media (Immediate) | 0.38 |
|  |  | Media + NaOH (Immediate) | 1.25 |
|  |  | Media + DMSO (Immediate) | 1.38 |
|  |  | S1 (Immediate) | 9.88 |
|  |  | S1 (Immediate) | 12.13 |
|  |  | Media (Overnight) | 0.21 |
|  |  | Media + NaOH (Overnight) | 1.29 |
|  |  | Media + DMSO (Overnight) | 1.50 |
|  |  | S1 (Overnight) | 2.36 |
|  |  | S1 (Overnight) | 0.00 |
|  | Donor D | Media (Immediate) | 1.29 |
|  |  | Media + NaOH (Immediate) | 1.29 |
|  |  | Media + DMSO (Immediate) | 0.43 |
|  |  | S1 (Immediate) | 3.43 |
|  |  | S1 (Immediate) | 4.71 |
|  |  | Media (Overnight) | 0.00 |
|  |  | Media + NaOH (Overnight) | 1.50 |
|  |  | Media + DMSO (Overnight) | 1.50 |
|  |  | S1 (Overnight) | 4.50 |
|  |  | S1 (Overnight) | 3.00 |

*All conditions were plated at 100K cells per well*

** Indicates representative donor presented in figure*

**Table S8. Individual donor stimulation index IFNγ responses for Figure 7**

| Figure | Donor ID | Condition | Stimulation index (SI) as compared to medias |
| --- | --- | --- | --- |
| Figure 7 | *Donor A | Media | 0.17 |
|  |  | Media | 1.39 |
|  |  | Media + NaOH | 0.87 |
|  |  | Media + DMSO | 1.57 |
|  |  | S1 | 10.96 |
|  |  | S1 | 13.04 |
|  | Donor D | Media | 0.33 |
|  |  | Media | 1.33 |
|  |  | Media + NaOH | 1.67 |
|  |  | Media + DMSO | 0.67 |
|  |  | S1 | 5.33 |
|  |  | S1 | 6.33 |

*All conditions were plated at 100K cells per well*

** Indicates representative donor presented in figure*

**Table S9. Common HLA-DRB alleles in Australian Caucasian populations**

| **AUS-NSW(891)** | **“AUS-SOUTH+EAST (1210)** |
| --- | --- |
| DRB1*04 | DRB1*04 |
| DRB1*15 | DRB1*15 |
| DRB1*03 | DRB1*07 |
| DRB1*07 | DRB1*03 |
| DRB1*13 | DRB1*01 |
| DRB1*01 | DRB1*13 |
| DRB1*11 | DRB1*11 |
| DRB1*14 |  |
| DRB1*08 |  |
| DRB1*12 |  |
| DRB1*10 |  |
| DRB1*16 |  |
| DRB1*09 |  |

Table generated using data from the Allele Frequencies in Worldwide populations database (RRID: SCR_007259) (1)

**Table S10. NaOH (1M), and DMSO concentrations within each respective priming, and endpoint control and stimulation conditions.**

| **Priming Condition** | 1M NaOH concentration (v/v) during 1 hour prime | 1M NaOH concentration (v/v) during post-prime | DMSO concentration (v/v) during 1 hour prime | DMSO concentration (v/v) during post-prime |
| --- | --- | --- | --- | --- |
| S1 pool | 0.05% | 0.0125% | 0.25% | 0.0625% |
| CMV-495 | Nil | Nil | Nil | Nil |
| Wuhan SARS-CoV-2 S-protein | Nil | Nil | Nil | Nil |
|  |  |  |  |  |
| **ELISpot/Fluorospot condition** | 1M NaOH concentration (v/v) during ELISpot/Fluorospot | | DMSO concentration (v/v) during ELISpot/Fluorospot | |
| Media | Nil | | Nil | |
| Media + NaOH | 0.49% | | Nil | |
| Media + DMSO | Nil | | 0.375% | |
| CMV-495 | Nil | | Nil | |
| S1 pool | 0.05% | | 0.25% | |
| PPD | Nil | | Nil | |
| SP_131-145 | 0.025% | | Nil | |
| SP_166-180 | 0.025% | | Nil | |
| XBB | Nil | | Nil | |

v/v indicates the concentration as the percentage of volume of NaOH or DMSO per total mixture volume present within the priming, control or stimulation wells.


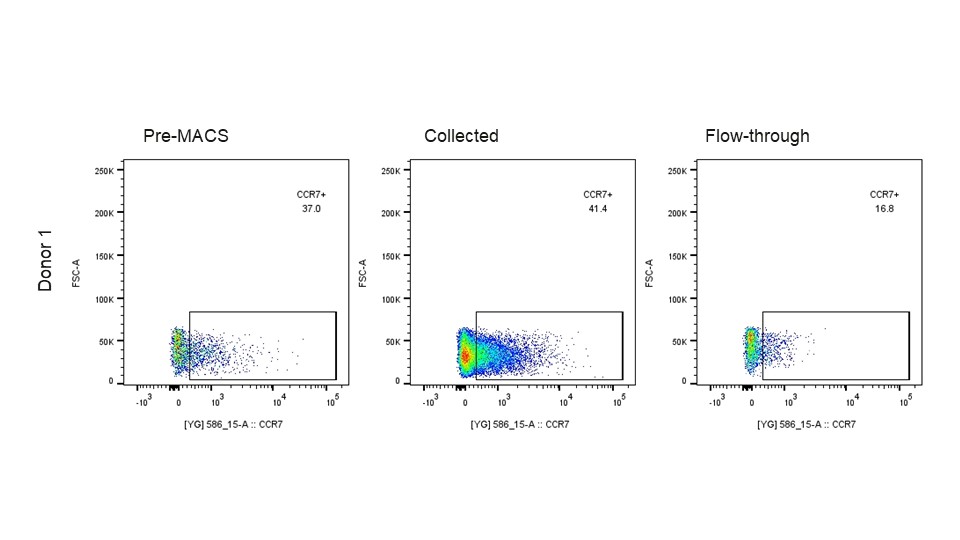


**Figure S1. FACS plots of the CCR7+ cell population from the various fractions collected during MACS depletion.** Pre-MACS unlabelled, and post-MACS collected (CCR7+) and Flow-through (CCR7 depleted) cell fractions were analysed for CCR7-PE staining as a proportion of all live PBMCs. Demonstrating a 56% reduction in CCR7+ cells as a proportion of all live PBMCs.

**Table S11. Raw counts and viability for complete and CCR7+ depleted fractions post-culture**

|  | **Live cells/mL** | **Dead cells/mL** | **Viability** |
| --- | --- | --- | --- |
| **Donor A Complete fraction count 1** | 1.82x10^6^ | 0.455 x10^6^ | 80% |
| **Donor A Complete fraction count 2** | 2.13 x10^6^ | 1.00 x10^6^ | 68% |
| **Donor A CCR7 depleted fraction count 1** | 0.419 x10^6^ | 0.291 x10^6^ | 59% |
| **Donor A CCR7 depleted fraction count 2** | 0.460 x10^6^ | 0.088 x10^6^ | 84% |

**References**

1. Gonzalez-Galarza FF, McCabe A, Santos E, Jones J, Takeshita L, Ortega-Rivera ND, et al. Allele frequency net database (AFND) 2020 update: gold-standard data classification, open access genotype data and new query tools. Nucleic Acids Res. 2020;48(D1):D783-D8.
